# Full‐Stack Architectures for Intelligent Brain‐Computer Interfaces

**DOI:** 10.1002/advs.75838

**Published:** 2026-05-28

**Authors:** Hee Kyu Lee, Hyun Bin Kim, Sang Uk Park, Janghoon Joo, Jinhong Min, Geumbee Lee, Joohoon Kang, Hyoyoung Jeong, Jae‐Young Yoo, Sang Min Won

**Affiliations:** ^1^ Department of Electrical and Computer Engineering Sungkyunkwan University Suwon Republic of Korea; ^2^ Department of Chemical and Biomolecular Engineering Yonsei University Seoul Republic of Korea; ^3^ School of Chemical Engineering and Applied Chemistry Kyungpook National University Daegu Republic of Korea; ^4^ Department of Electrical and Computer Engineering University of California Davis CA USA; ^5^ Department of Semiconductor Convergence Engineering Sungkyunkwan University Suwon Republic of Korea

**Keywords:** brain–computer interface, chronic signal stability, closed‐loop BCI, electrode–tissue interface, neural decoding, neural interface, wireless neural recording

## Abstract

Brain–computer interfaces (BCIs) have made consistent advances in supporting motor and communication functions; nevertheless, their adoption in everyday environments remains constrained by enduring challenges, including chronic instability at the electrode–tissue interface, motion‐induced artifacts, inter‐user variability, and strict power and bandwidth limitations. To address these issues, recent work has increasingly focused on system‐level innovations encompassing electrode design, wireless communication strategies, and neural decoding algorithms. At the interface level, enhancements in electrochemical performance and mechanical compliance improve long‐term electrode–tissue coupling and help maintain signal integrity during naturalistic movement. For signal acquisition and transmission, miniaturized front‐end electronics and energy‐efficient telemetry architectures enable higher channel counts while minimizing power consumption and optimizing bandwidth utilization. In parallel, decoding approaches have evolved from static, feature‐based pipelines toward adaptive machine‐learning and deep‐learning methods that are more resilient to nonstationary neural signals and capable of supporting low‐latency, closed‐loop operation. This review consolidates findings from contemporary preclinical and human studies to provide a comprehensive perspective on system‐level engineering strategies for practical BCI technologies, emphasizing neural interface architecture and system‐design approaches that enhance signal stability and real‐world usability, while also identifying emerging design paradigms that may facilitate next‐generation BCIs with improved scalability and broader practical impact.

## Graphical abstract

System‐level overview of brain–computer interfaces (BCIs), illustrating the integration of neural signal acquisition, wireless transmission, and adaptive decoding. Advanced electrode, tissue interfaces, energy‐efficient communication, and robust algorithms collectively enable stable signal quality, real‐time processing, and closed‐loop operation, highlighting key engineering strategies for scalable and practical BCI systems in real‐world environments.

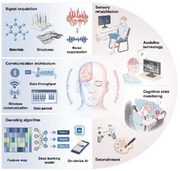

## 1 Introduction

The central nervous system implements higher‐order functions such as cognition, motor control, and sensory processing through spatiotemporal patterns of electrical activity generated across neural circuits extending from the cerebral cortex to the spinal cord [1]. Action potentials and local field potentials (LFPs) generated by neuronal populations manifest as measurable electrical signals at the electrode interface [2, 3, 4, 5]. This provides the theoretical basis for quantitatively decoding neural activity and interfacing it with external systems. Brain–Computer Interfaces (BCIs) are technologies that enable bidirectional information exchange between the human nervous system and machines [6, 7, 8]. They operate by recording and decoding central nervous system signals in real time to generate digital outputs that control external devices or provide neural stimulation.

Clinically, BCIs have demonstrated the ability to restore or assist motor and communication functions in patients with spinal cord injury (SCI) [9, 10, 11], amyotrophic lateral sclerosis (ALS) [12, 13, 14], and stroke [15, 16]. Notable demonstrations include robotic arm control and text entry at rates exceeding several tens of characters per minute by decoding motor cortical signals from implanted microelectrode arrays (MEAs) in individuals with tetraplegia [17, 18, 19]. These results establish the clinical feasibility and therapeutic potential of BCI systems. More recently, BCI applications have expanded beyond medical use cases to non‐clinical domains, including cognitive state monitoring [20], neurorehabilitation [21], and augmented (AR)/virtual reality (VR)‐based interfaces [22, 23].

**FIGURE 1:**
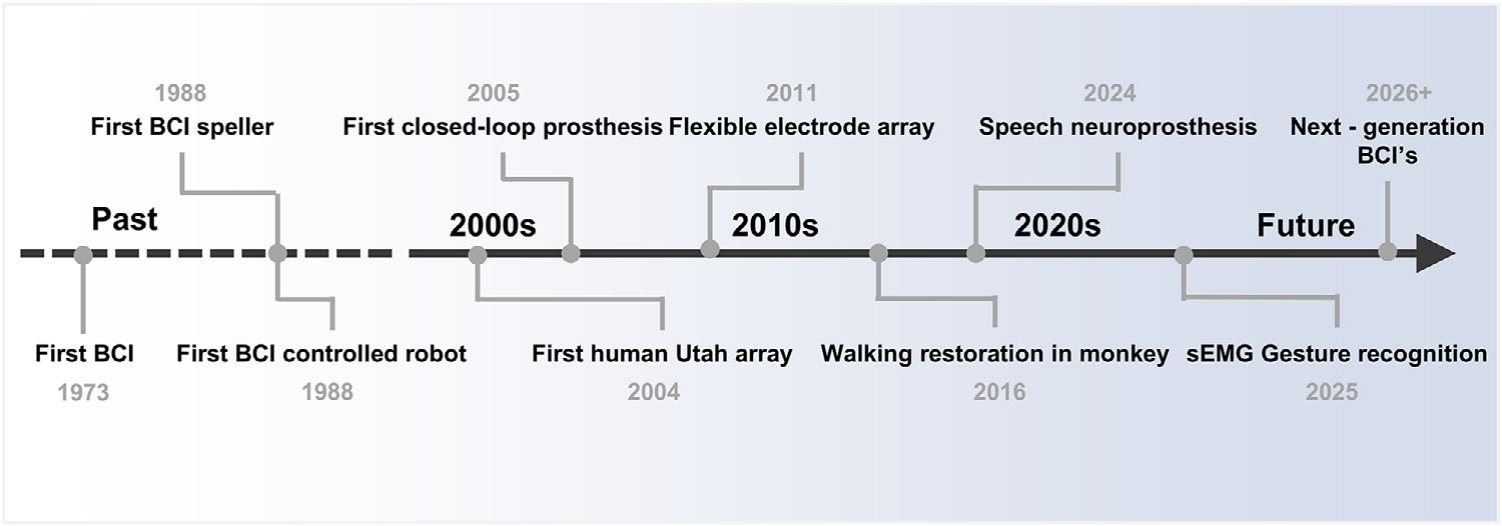
Evolution of BCI technologies. Timeline illustrating the progression of BCI technologies across decades, highlighting key milestones in both foundational research and applied neuroengineering. In the 1970's, the first BCI systems is demonstrated, establishing the feasibility of direct brain signal decoding [24]. In the 1980's, the introduction of the P300 speller demonstrates the use of event‐related potentials for communication [36], while early BCI‐controlled robotic systems mark initial efforts toward external device control [37]. In the 2000's, the first human implantation of the Utah array enables high‐resolution intracortical recordings [30], significantly advancing neural interfacing capabilities, while real‐time intracortical control of robotic arms is demonstrated, marking a major breakthrough in translating neural activity into continuous motor outputs [38, 39]. During the 2010's, the field sees the introduction of flexible high‐density electrode arrays, enabling improved spatial resolution and scalability [40], alongside the growing adoption of wireless BCI systems that enable untethered neural recording and real‐world deployment. Furthermore, closed‐loop neuroprosthetic applications expand to demonstrate functionalities such as walking restoration in non‐human primates [41]. In the 2020's, rapid advances in decoding, including the emergence of real‐time speech‐to‐text neuroprosthetic interfaces, enable direct translation of neural activity into continuous language output [42], alongside a broader shift toward wearable and non‐invasive systems such as surface electromyography (sEMG)‐based gesture recognition, as well as continued progress in flexible electronics [43]. Looking toward the future, next‐generation BCIs aim to integrate high‐resolution recording, wireless communication, and real‐time AI‐driven decoding to enable scalable, adaptive, and clinically viable neurotechnologies.

This development is rooted in a long trajectory of BCI research that began with electroencephalogram (EEG)‐based cursor control experiments in the early 1970s [24]. Since then, advances in microelectronics and computational neuroscience have driven the development of increasingly diverse modalities, ranging from invasive MEAs to non‐invasive high‐density systems [25, 26, 27, 28, 29]. Since the 2000s, advances in multichannel recording miniaturization, wireless data transmission, and machine learning‐based decoding have driven the evolution of BCI technologies [30, 31, 32, 33, 34, 35]. These developments have enabled a transition from laboratory‐based proof‐of‐concept demonstrations to clinical level implementations (Figure 1).

Despite these advances, significant challenges remain for the deployment of BCIs in everyday environments. Key limitations include reduced biocompatibility at the electrode–tissue interface and the associated degradation of signal quality, decoding instability caused by inter‐user variability and temporal fluctuations in neural signals, and efficiency losses and data transmission bottlenecks in high‐density multichannel systems [44, 45, 46, 47, 48]. Addressing these challenges requires integrated progress across materials science, systems engineering, and adaptive signal processing methodologies [49, 50].

**FIGURE 2:**
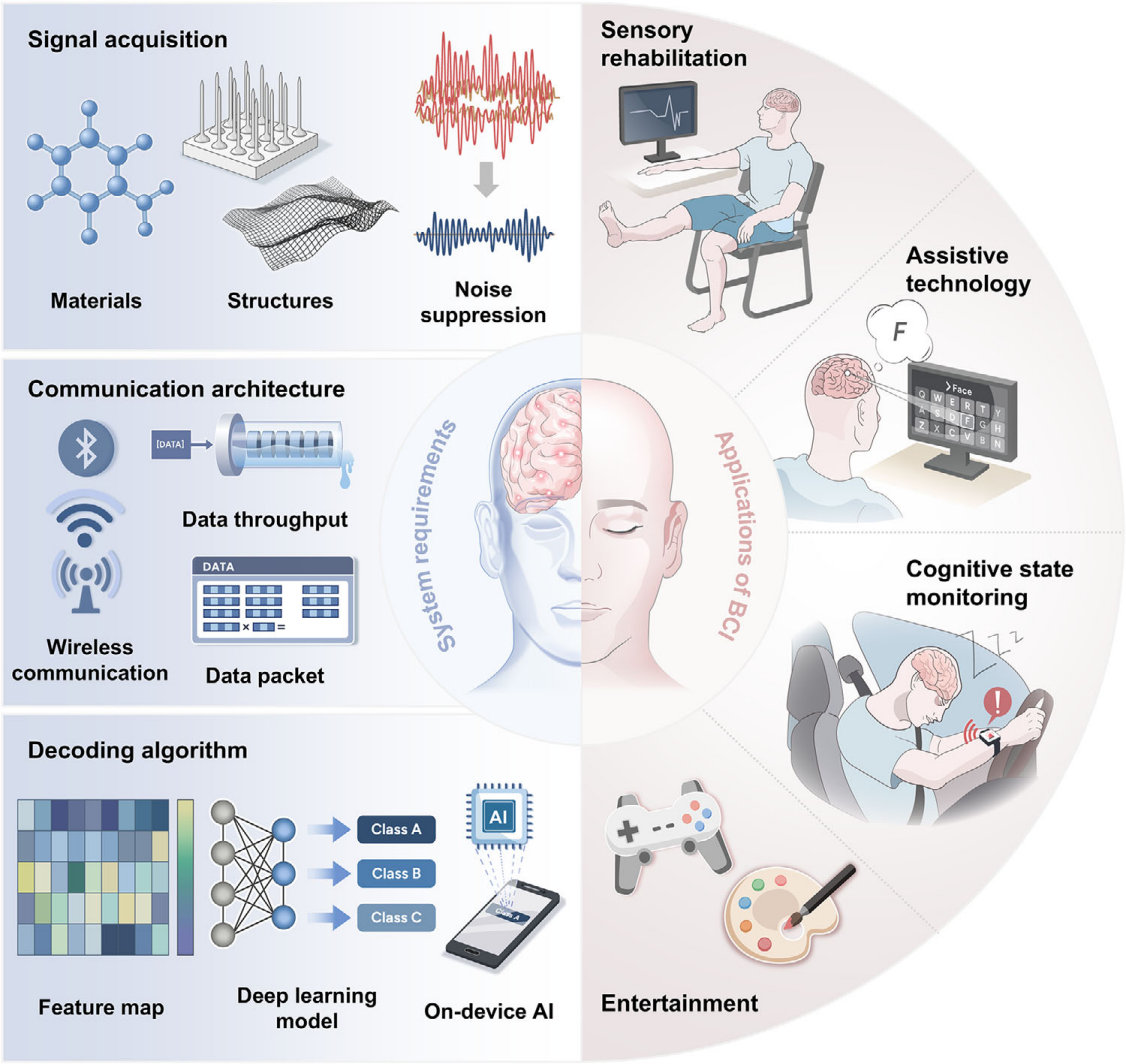
Overview of BCI systems.

This review provides a comprehensive overview of system‐level engineering strategies that support the practical deployment of BCI technologies (Figure 2). We examine recent preclinical and early human studies that advance neural interface technologies. Our focus is on material and structural strategies for improving signal stability, low‐power and high‐bandwidth wireless architectures, and adaptive decoding methods that compensate for signal variability under real‐world conditions. By integrating perspectives across materials, electrodes, systems, and algorithms, this review identifies key requirements and remaining technical challenges for achieving reliable, real‐time, and everyday‐use BCI platforms.

## 2 Technologies for Improving Intrinsic Neural Signal Quality

The performance of neural recording technologies is fundamentally constrained by the electrode's mechanical properties, electrochemical stability, spatial resolution, long‐term signal reliability, and channel scalability [51, 52, 53, 54, 55, 56, 57]. Conventional clinical electrodes have served as the foundation for capturing neural activity in both non‐invasive and invasive settings. In clinical diagnostics, for example, silver (Ag)/silver chloride (AgCl) cup electrodes placed on the scalp are routinely employed to record EEG signals, enabling detection of epileptiform discharges and coarse localization of pathological regions [58, 59]. During epilepsy surgery or tumor resection, platinum (Pt)/iridium (Ir) subdural grids record electrocorticography (ECoG) signals, allowing surgeons to delineate eloquent cortical areas and preserve critical motor and language functions [60, 61].

Although these clinical electrodes provide well‐established stability and reliability, they exhibit structural and electrochemical limitations that constrain their use in advanced applications. Rigid substrates and limited mechanical compliance create a mismatch with soft neural tissue, leading to micromotion‐induced signal instability during chronic implantation [62, 63, 64]. As electrode dimensions decrease to improve spatial resolution, the reduced electrode–tissue contact area leads to increased interfacial impedance, thereby reducing the signal‐to‐noise ratio (SNR) and limiting the detection of fine‐grained neural activity [65]. These electrical and mechanical constraints have motivated the development of next‐generation neural interfaces that address mechanical compatibility, electrochemical performance, and scalable recording capability.

To systematically address these challenges, neural interfaces can be understood in terms of three fundamental failure modes: (i) electrochemical limitations at the electrode–tissue interface, which limit efficient charge transfer and result in increased interfacial impedance; (ii) mechanical mismatch between rigid devices and soft neural tissue, which induces interfacial instability under chronic micromotion; and (iii) limited spatial sampling capability in three‐dimensional neural environments, which constrains the resolution and coverage of neural recordings. Based on this framework, the following subsections are organized according to the specific failure modes they address. Section 2.1 focuses on strategies for reducing interfacial impedance and enhancing electrochemical performance; Section 2.2 discusses approaches for mitigating mechanical mismatch and improving long‐term interfacial stability; and Section 2.3 addresses strategies for overcoming spatial sampling limitations through three‐dimensional (3D) electrode architectures.

**FIGURE 3:**
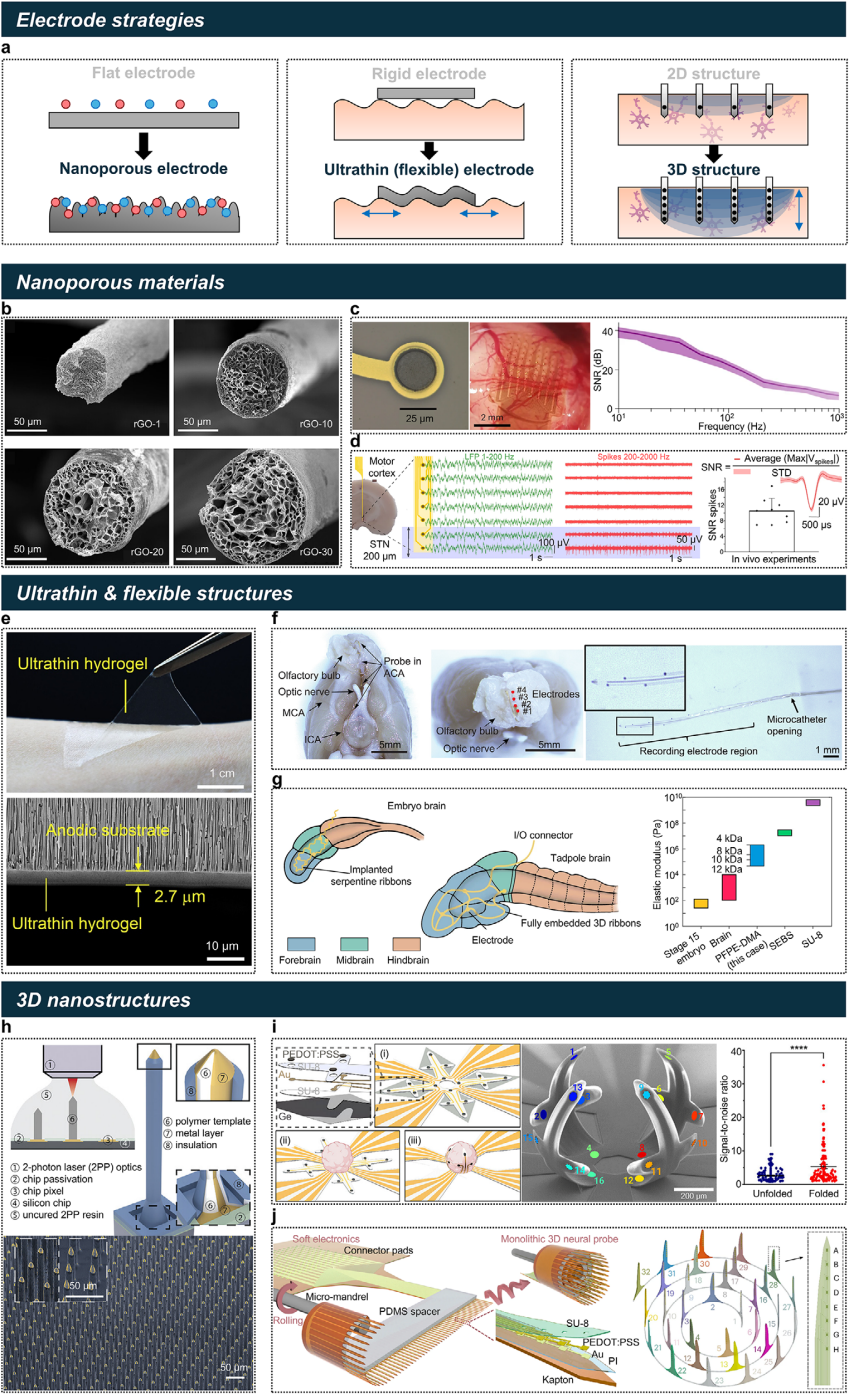
Advanced materials and architectures for neural interfaces. (A) Electrode design strategies for improving neural interface performance; (B) Reduced graphene oxide (rGO) microfibers with a tunable nanoporous architecture. Reproduced with permission [73]. Copyright 2025, American Chemical Society; (C) Miniaturized graphene‐based thin‐film microelectrode (diameter of 25 µm). Reproduced under terms of the CC‐BY license [74]. Copyright 2024, Springer Nature; (D) Microscale rGO electrodes implanted into the subthalamic nucleus for deep brain recording in Parkinsonian models. Reproduced under terms of the CC‐BY license [108]. Copyright 2025, Springer Nature; (E) Ultrathin nanomesh‐reinforced phase‐change gelatin hydrogel interface (thickness ∼ 2.7 µm) for conformal and stable biosignal monitoring. Reproduced with permission [114]. Copyright 2025, The American Association for the Advancement of Science; (F) Miniaturized ultraflexible micro‐endovascular neural probe implanted through sub‐100 µm vessels for in vivo neural recording. Reproduced with permission [77]. Copyright 2023, The American Association for the Advancement of Science; (G) Submicrometer‐thick mesh microelectrode array integrated during embryonic neural development for brain‐wide electrophysiological recording. Reproduced with permission [76]. Copyright 2025, Springer Nature; (H) Volumetric 3D microelectrode array fabricated by two‐photon polymerization for ultrahigh‐density neural interfacing. Reproduced under terms of the CC‐BY license [129]. Copyright 2024, John Wiley and Sons; (I) Self‐folded shell‐type 3D microelectrode array conformally enveloping a spherical neural organoid. Reproduced with permission [83]. Copyright 2025, John Wiley and Sons; (J) 3D neural probe formed by rolling‐of‐soft‐electronics from planar flexible electrodes. Reproduced with permission [84]. Copyright 2025, Springer Nature.

Figure 3A provides a conceptual overview of representative design strategies in the context of these failure modes. Specifically, electrochemical limitations can be mitigated by increasing the effective interfacial surface area using nanoporous and nanostructured electrodes, which reduce impedance and enhance charge storage capacity (CSC). Mechanical mismatch can be alleviated by ultrathin and flexible interfaces that enable conformal integration with neural tissue. Spatial sampling constraints can be addressed by 3D electrode architectures that enable volumetric neural recording. These approaches collectively define the design space of next‐generation neural interfaces and are discussed in detail in the following sections. To provide a quantitative perspective, key performance metrics across these strategies are summarized in Table 1.

**TABLE 1:** Quantitative comparison of representative neural electrode strategies.

Electrode strategies	Impedance (at 1 kHz)	CSC (mC/cm^2^)	Modulus of elasticity	In vivo duration	Key advantages	Limitation	References
Planar metal (Pt, Au)	∼100 kΩ–1 MΩ	< 20	78 GPa (Au) 172 GPa (Pt)	weeks – months (chronic)	Stable fabrication	High impedance, mechanical mismatch	[66, 67, 68, 69, 70]
Nanostructured materials	∼1–30 kΩ	∼50–400	kPa–MPa	days–months	Low impedance, high CSC	Stability issues	[71, 72, 73, 74, 75]
Ultrathin & ultraflexible design	∼10–500 kΩ	∼20–220	Pa–kPa	weeks – months	Mechanical compliance	Handling difficulty	[70, 76, 77, 78, 79]
3D electrode architecture	∼10–800 kΩ	∼20	kPa–MPa	weeks – months	High spatial resolution	Tissue damage	[75, 80, 81, 82, 83, 84]

### 2.1 Strategies for Reducing Interfacial Impedance and Enhancing Electrochemical Performance

Neural electrodes are designed to record neuronal electrical activity and to electrically stimulate neural tissue. However, electrochemical limitations at the electrode–tissue interface remain a major obstacle to achieving high‐fidelity recording and stimulation performance [51, 85]. As electrode dimensions are reduced to improve spatial resolution, the effective contact area correspondingly decreases, resulting in increased interfacial impedance and degraded SNR. Moreover, limited CSC and insufficient electrochemical stability further restrict reliable neural signal acquisition and stimulation. To overcome these limitations, recent studies have increasingly focused on nanoporous electrode architectures that enhance interfacial electrochemical performance by increasing the electrochemically active surface area and facilitating ionic transport.

Micro‐ and nanoscale porosity reduces interfacial impedance and associated thermal and polarization noise at the electrode–tissue interface, leading to higher SNR [86, 87, 88]. Recent studies demonstrate nanoporous structures across diverse material systems, including nanoporous Pt [89, 90, 91], carbon nanotube (CNT) arrays and fibers [92, 93, 94, 95], nanoporous metal oxides [72, 96, 97, 98, 99, 100], and laser‐induced graphene [101, 102]. For example, uniformly distributed nanoporous Pt films exhibit the lowest impedance and the highest SNR among the tested morphologies [90]. Similarly, CNT fiber electrodes exhibit substantially enhanced electrochemical performance, including low impedance and high interfacial charge capacity, supporting stable, high‐SNR neural recording [94]. These characteristics position nanoporous electrodes as highly efficient material platforms for next‐generation neural interfaces.

Among these, nanostructured carbon‐based materials incorporating graphene are widely adopted for neural electrodes owing to their favorable combination of electrochemical performance, mechanical robustness, flexibility, and biocompatibility [103]. One example leverages reduced graphene oxide (rGO) microfibers with tunable porous architectures, achieving ultralow impedance and markedly enhanced CSC (Figure 3B) [73]. These properties are enabled by controlled wet‐spinning processes that increase the electrochemically active surface area while maintaining mechanical integrity. Nanoporous electrode materials when miniaturized to the micrometer scale enable high–spatial‐resolution neural recording while maintaining high electrode density, which is directly associated with improved neural signal decoding performance.

One recent example presents a graphene‐based thin‐film electrode, achieving ∼ 25 kΩ impedance at 1 kHz at a 25 µm diameter (Figure 3C) [74]. This performance arises from a defect‐rich, nanoporous graphene architecture that mitigates impedance increases during miniaturization, enabling dense electrode arrays with preserved signal quality [104, 105]. In vivo cortical recordings demonstrate LFP SNRs exceeding 10 dB and power spectral density (PSD) values approaching ∼ 40 dB at 10 Hz, outperforming conventional Pt‐based micro‐electrocorticography (µECoG) electrodes [54, 106]. Beyond cortical applications, miniaturized rGO electrodes also provide advantages for deep brain interfaces, where conventional deep brain stimulation (DBS) electrodes are limited by millimeter‐scale contacts and tissue damage [107]. rGO electrodes implanted into the subthalamic nucleus record mean spike SNR values of 10.4 ± 3.2 and reliably resolved elevated firing rates in Parkinsonian models, highlighting their suitability for microscale neural recording and biomarker‐driven deep brain neuromodulation (Figure 3D) [108].

### 2.2 Strategies for Mitigating Mechanical Mismatch and Enhancing Interfacial Stability

Mechanical mismatch between soft neural tissue and rigid electrode materials remains a fundamental barrier to long‐term interfacial stability. Tissue has a Young's modulus in the 1–10 kPa range, whereas conventional metal electrodes exhibit stiffness in the GPa range, resulting in the implanted electrodes to generate significant interfacial stress both during insertion and subsequently during chronic micromotion [109]. This mechanical mismatch generates persistent interfacial shear stress, which triggers chronic inflammation, glial scarring, and fibrotic encapsulation, ultimately increasing impedance and degrading signal stability [57, 110]. To mitigate these challenges, recent neural interface strategies increasingly adopt mechanically compliant designs based on ultrathin and ultraflexible structures [57, 111].

Ultrathin and ultraflexible structural designs stabilize neural signal quality by improving mechanical compliance matching at the electrode–tissue interface. As bending stiffness decreases rapidly with thickness, ultrathin electrodes can achieve tissue‐level effective stiffness even when fabricated from stiff materials [64, 112, 113]. This mechanical compliance enables co‐deformation with neural tissue, reducing interfacial shear stress and maintaining stable electrode–tissue contact. As a result, ultrathin electrodes suppress impedance fluctuations and exhibit reduced impedance drift during long‐term implantation, supporting sustained neural decoding performance [57, 113].

Building upon this thickness‐driven compliance, hydrogel‐based interfaces further enhance interfacial stability by introducing tissue‐like mechanical properties. When engineered at micrometer‐scale thicknesses, ultrathin hydrogels preserve extremely low bending stiffness while improving conformal contact and environmental robustness. For example, nanomesh‐reinforced phase‐change gelatin hydrogels with a thickness of 2.7 µm demonstrate improved adhesion and stable signal acquisition under daily mechanical deformation, enabling continuous wireless biosignal monitoring for up to 8 days (Figure 3E) [114].

In addition to improving interfacial stability, ultraflexible interface designs also minimize tissue damage and attenuate inflammatory responses associated with penetrating electrodes during in vivo endovascular implantation and electrophysiological recording. A recent study demonstrates miniaturized, ultraflexible micro‐endovascular neural probes that can be implanted through sub‐100 µm vessels in rodents without causing damage to brain tissue or vascular structures (Figure 3F) [77]. In vivo electrophysiological recordings show selective measurement of LFPs and single‐neuron spikes in the cortex and olfactory bulb. Furthermore, histological analyses confirm minimal immune response and excellent long‐term stability at the flexible probe–vessel–brain interface.

Beyond static or adult tissue environments, ultraflexible neural interfaces have been extended to accommodate continuous tissue growth during development. A recent study exploits embryonic morphogenesis to achieve seamless, brain‐wide integration of a submicrometer‐thick mesh MEA by positioning it on the neural plate, whereupon neural tube formation drives its 3D internalization (Figure 3G) [76]. The mesh, fabricated on perfluoropolyether–dimethacrylate (PFPE–DMA) substrate with a low elastic modulus (∼ 0.3 MPa with 8‐kDa molecular weight), maintains its spatial position while conformally following neural plate–to–neural tube transformation and subsequent brain growth. Enabled by extremely low bending stiffness, a serpentine interconnect architecture, and low effective interfacial stress, the interface accommodates large morphological changes without inducing mechanical damage. This growth‐adaptive ultraflexible platform supports stable, brain‐wide electrophysiological recording with single‐unit resolution across developmental stages, collectively establishing ultraflexible neural interfaces as a viable platform for sustained neural recording across diverse and dynamically evolving biological environments.

### 2.3 Strategies for Enhancing Spatial Sampling and Recording Density

Neural tissue is inherently three‐dimensional, motivating electrode architectures that overcome the geometric limitations of planar MEAs. 3D electrode architectures expand spatial sampling capability by improving geometric alignment with distributed neuronal populations and enabling volumetric recording coverage across cortical depth and tissue volume [115, 116, 117, 118]. Conventional platforms such as Michigan probes [119, 120] and Utah arrays [121, 122, 123] established depth‐resolved multichannel recording; however, their rigid silicon‐based geometries impose fixed layouts, limited adaptability, and increased tissue damage, constraining long‐term stability and scalable 3D interfacing [124, 125, 126].

Recent efforts have focused on direct 3D fabrication strategies that realize volumetric electrode architectures. Such printing approaches enable freeform geometric design with programmable shank height, diameter, and spacing, allowing region‐specific and depth‐targeted neural recording. Techniques including metal nanoparticle printing [82], liquid metal extrusion [127], and digital light processing‐based printing [128] demonstrate customizable 3D electrode arrays without reliance on planar constraints. Among these, two‐photon polymerization (2PP) provides submicrometer‐resolution fabrication of complex 3D microelectrode structures directly on microelectronic substrates, enabling ultrahigh‐density volumetric arrays (> 6600 channels at ∼ 35 µm pitch) while maintaining compatibility with complementary metal–oxide–semiconductor (CMOS)‐based neural interfaces (Figure 3H) [129].

In parallel, an alternative class of strategies realizes 3D neural interfaces through 2D‐to‐3D structural transformation, leveraging material‐intrinsic stress and mechanically induced deformation. Self‐folding approaches based on differential strain in bilayer materials enable planar microfabricated electrodes to spontaneously transform into shell‐type 3D MEAs that conformally envelop spherical tissues [130]. For example, self‐folding of SU‐8 bilayers form spherical cages that uniformly distribute 16 electrodes around an organoid (Figure 3I) [83]. This geometry enables 360° surface contact and whole‐surface sampling of electrical propagation from a single organoid and reconstruction of quantitative 3D isochrone maps and conduction‐velocity vector fields using simultaneous multichannel recordings.

Beyond material‐intrinsic approaches, a range of mechanical transformation strategies, including curving [131, 132, 133, 134, 135], buckling [136, 137, 138, 139], and folding [80, 140, 141, 142], have been integrated with microfabrication to deploy high‐resolution planar electrode patterns into deterministic 3D configurations [143]. A representative example applies the kirigami concept to surface‐micromachined flexible MEAs, enabling deterministic 2D‐to‐3D conversion with high structural yield (∼ 98%) []. Another representative strategy is rolling‐of‐soft‐electronics (ROSE), which converts planar flexible electrodes into monolithic cylindrical 3D neural probes via controlled rolling (Figure 3J) [84]. The final probe geometry is predefined by planar design parameters such as shank number, shank pitch, and polydimethylsiloxane (PDMS) spacer thickness, enabling architectures with up to 64 shanks and 256 channels. This rolling framework offers greater geometric versatility than pattern‐dependent approaches by allowing systematic variation of loop number, layering, and radial shank distribution within a single fabrication scheme. In vivo studies in rodents and non‐human primates demonstrate stable single‐unit recordings and 3D mapping of cortical activity, while chronic implantation over 5 weeks confirmed sustained signal stability. Across direct fabrication and structural transformation approaches, these strategies establish 3D electrode architectures as a versatile and scalable solution for high‐density, volumetric neural recording beyond the geometric constraints of planar designs.

## 3 Strategies for Artifact Mitigation

Noise interference arising from mechanical, biological, electrical, and positional instabilities degrades neural signals at the electrode–tissue interface and limits decoding performance [145, 146]. Electrode micromotion and chronic mechanical coupling induce impedance fluctuations, motion artifacts [147], and glial encapsulation [146, 147], while electrical interference directly contaminates recording circuitry [148, 149, 150]. In parallel, electrode displacement driven by tissue dynamics and biological remodeling progressively reduces spatial coupling to target neuronal populations, leading to long‐term sensitivity loss [57, 146, 151]. Accordingly, recent research has moved beyond material‐ and structure‐level optimization toward complementary system‐level noise mitigation strategies. In this section, we systematically review these noise suppression strategies and analyze how each approach addresses specific noise mechanisms at the system level.

### 3.1 Wireless Neural Recording for Stable Long‐Term Operation

Fully wireless neural recording systems fundamentally improve long‐term signal stability by eliminating tethered cables that induce mechanical stress, impedance fluctuation, and motion‐related artifacts at the electrode–tissue interface [152, 153]. Shortened interconnect paths further reduce parasitic capacitance and inductance, structurally suppressing power‐line interference and high‐frequency electromagnetic coupling [154]. These effects mitigate dominant noise sources that accumulate over extended operation, enabling more reliable chronic neural recording. Experimental comparisons directly demonstrate that wired systems employing ∼ 1 m cables exhibit power‐line noise exceeding 5 µV/√Hz, whereas miniaturized wireless platforms (9 × 7 × 5 mm^3^) achieve noise levels below 100 nV/√Hz resulting in substantially improved SNR [155]. Importantly, this noise suppression persists under dynamic conditions, indicating that mechanical decoupling is the primary stabilizing mechanism [156, 157].

**FIGURE 4:**
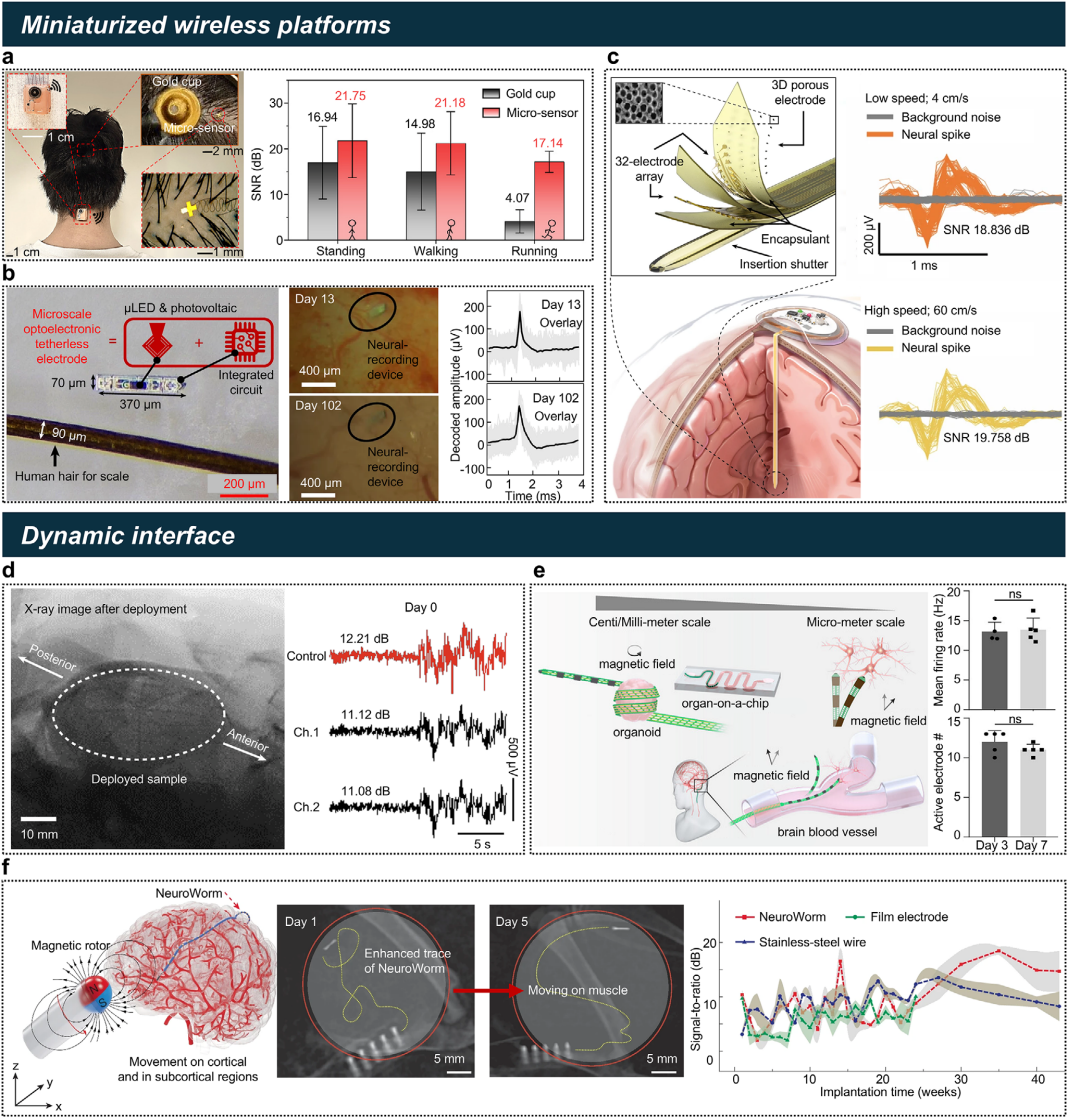
Wireless and dynamic neural recording systems. (A) Ultra‐compact wireless EEG microsensor designed for sub‐millimeter placement between hair strands for motion‐resilient noninvasive neural recording. Reproduced with permission [158]. Copyright 2025, National Academy of Sciences; (B) Ultraminiaturized photovoltaic–light‐emitting diode–based fully implantable neural recording microsystem enabling battery‐free wireless operation. Reproduced with permission [159]. Copyright 2025, Springer Nature; (C) Fully implantable wireless neural recording platform integrated beneath the scalp of a non‐human primate for chronic recording during free behavior. Reproduced with permission [160]. Copyright 2024, Springer Nature; (D) Shape‐memory polymer–based electronic tent interface that autonomously deploys in situ after implantation. Reproduced with permission [166]. Copyright 2024, Springer Nature; (E) Magnetically actuated neural probe enabling post‐implantation positional adjustment within three‐dimensional tissue. Reproduced with permission [168]. Copyright 2025, John Wiley and Sons; (F) Magnetically guided flexible bioelectronic fiber capable of post‐implantation locomotion for dynamic neural interfacing. Reproduced with permission [169]. Copyright 2025, Springer Nature.

In noninvasive settings, an ultra‐compact wireless EEG microsensor designed for sub‐millimeter placement between hair strands (< 1 mm) demonstrates robust signal fidelity during prolonged motion (Figure 4A) [158]. The serpentine interconnects mechanically isolate the sensing electrodes from external perturbations, preserving electrode–skin coupling during continuous activity. The device shows negligible SNR degradation after 12 hours of use, even during running. In contrast, gold cup electrodes exhibit severe motion artifacts, rapid SNR loss, and frequent detachment after prolonged wear, limiting long‐term recording. These results highlight wireless miniaturization and mechanical decoupling as key enablers of stable noninvasive neural recording.

For invasive neural interfaces, transcutaneous leads introduce infection risk, micromotion‐induced tissue responses, and long‐term contact instability. Fully implantable wireless systems eliminate these limitations by removing lead‐related noise sources. A representative example is an ultraminiaturized photovoltaic–light‐emitting diode (PVLED)‐based neural recording microsystem (Figure 4B) [159]. With dimensions of approximately 370 µm × 70 µm, the device operates without batteries or radio frequency (RF) links, instead using optical power and data transmission. The system achieves an input‐referred noise of 14.8 µV_r.m.s._ and maintains stable neural recordings for up to 365 days in awake mice, directly demonstrating year‐scale chronic signal reliability.

Scaling fully implantable wireless systems to large‐animal models is critical for validating long‐term performance under complex, naturalistic behaviors. In non‐human primates, a fully implantable neural recording platform concealed beneath the scalp achieves stable chronic operation through optimized electromagnetic coupling (Figure 4C) [160]. The system integrates a 13.56 MHz receiver coil with ferrite flux concentrators and electromagnetic shielding, enhancing power‐transfer efficiency while suppressing motion‐ and environment‐induced interference. Stable SNR of 18.8 dB and 19.8 dB are maintained during slow (4 cm/s) and fast (60 cm/s) movement, respectively, confirming that wireless, battery‐free operation supports consistent neural signal quality during extended free behavior.

### 3.2 Dynamic Neural Interfaces

While recent advances in ultrathin and mechanically compliant neural interfaces have significantly improved mechanical matching at the electrode–tissue interface, these approaches primarily address mechanical stability rather than spatial positioning. Conventional static electrodes lack post‐implantation adjustability, leading to progressive electrode–neuron decoupling caused by tissue micromotion, growth, and anatomical variability, which degrades signal amplitude and increases noise over time [161, 162]. To address these limitations, recent studies have introduced dynamically controllable neural interfaces that enable post‐implantation deployment or repositioning, thereby maintaining spatial alignment with target neural populations and suppressing spatially induced noise [163, 164].

Shape‐memory polymer‐based interfaces provide minimally invasive implantation followed by autonomous in situ deployment, enabling stable long‐term contact [163, 165]. Stimuli‐responsive expansion or structural transformation enables the interface to conform closely to the geometry of the target tissue, establishing intimate contact and thereby enhancing the accuracy of signal transmission. An electronic tent–type interface incorporating poly(lactide‐*co*‐*ε*‐caprolactone)–poly(lactic‐*co*‐glycolic acid) (PLCL–PLGA) shape‐memory layers deploys radially at physiological temperature after insertion (Figure 4D) [166]. In vivo canine experiments demonstrate stable 16‐channel ECoG recordings from molybdenum (Mo) electrodes over extended periods, confirming sustained cortical contact and signal fidelity after deployment. The Mo electrodes initially exhibited an SNR of 11.38 ± 1.61 dB, which is comparable to that of control Pt electrodes (11.78 ± 1.09 dB), indicating equivalent early‐stage recording performance following deployment. The deployment strategy based on shape‐memory behavior offers predictable and smooth expansion after insertion; however, its adaptability to spatial drift over time or dynamically changing target locations may be limited.

To further enhance post‐implantation positional control, magnetically actuated neural interfaces have been developed to enable active repositioning. Magnetic actuation using external fields enables remote and real‐time device steering, offering a versatile strategy for dynamic positional control in flexible bioelectronics. In one study, nano‐magnetic ink printing was employed to integrate magnetic nanoparticles into compliant neural interfaces, imparting sufficient magnetic responsiveness while preserving mechanical flexibility [167]. The resulting Mag‐N‐Probe (Magnetically guided Neural‐interfacing Probe) achieves sub‐micrometer positioning precision while supporting millimeter‐to‐centimeter scale navigation within complex three‐dimensional environments (Figure 4E) [168]. Longitudinal recordings at day 3 and day 7 show consistent spike activity, with no significant changes in mean firing rate or the number of active electrodes. The average SNR remain stable at ∼ 5.6 (14.8 dB), confirming sustained electrode–tissue coupling and reliable neural recording over multiple days. While this approach enables precise and active repositioning compared to passive deployment strategies, its operation is primarily limited to localized adjustment.

Building upon these advances, fully mobile bioelectronic systems have been introduced to enable continuous and large‐scale repositioning within tissue over extended periods. Dynamic repositioning over longer timescales is further demonstrated by NeuroWorm, a flexible bioelectronic fiber capable of magnetically guided locomotion within tissue (Figure 4F) [169]. The platform is formed by rolling an ultrathin flexible bioelectronic film into a 1D microfiber, yielding a small diameter and low bending stiffness that mitigate insertion‐induced damage and chronic tissue response. Magnetic actuation enables post‐implantation repositioning to access evolving functional hotspots without reimplantation. In vivo studies demonstrated stable electromyography (EMG) recordings for over 43 weeks, with minimal fibroblast encapsulation observed even after 54 weeks. These results highlight magnetically movable implants as an effective strategy for long‐term stable recording beyond static implantation.

### 3.3 Signal Processing

Despite advances in fully wireless architectures and dynamically adjustable interfaces, residual artifacts remain unavoidable in practical BCI operations. Even in cable‐free systems, electrode–skin slip, package micromotion, and transient impedance changes can induce baseline drift and broadband bursts, while myogenic activity and environmental electromagnetic interference continue to contaminate recordings [170]. In online control, these disturbances destabilize classifier confidence, increasing false detections and output jitter. Accordingly, system‐level signal processing complements hardware strategies by providing real‐time mechanisms to detect, suppress, and tolerate artifacts, spanning front‐end acquisition conditioning and downstream digital processing under latency, power, and telemetry constraints [171, 172].

At the analog front end (AFE), artifact mitigation is achieved through hardware‐level conditioning that stabilizes the input before analog‐to‐digital converter (ADC) sampling and any microcontroller‐level processing. The input stage suppresses predictable common‐mode interference by using an instrumentation amplifier with high common‐mode rejection and an appropriate reference scheme, which reduces power‐line pickup and ambient electromagnetic coupling at the electrodes [173, 174]. An analog anti‐alias low‐pass filter placed ahead of the ADC attenuates out‐of‐band components that would otherwise fold into the neural band during sampling [175, 176]. In addition, mechanically stable, low‐impedance electrode interfaces and local buffering or active‐electrode circuits reduce sensitivity to transient impedance changes and contact disturbances [173, 177, 178]. These measures constrain interference early in the signal chain, reducing downstream processing burden and limiting artifact propagation into decoding decisions.

Downstream digital processing in practical BCI systems typically relies on a small set of widely adopted modules that are robust and compatible with real‐time control. Sampled neural signals are commonly standardized through digital filtering, including task‐appropriate band‐pass filtering and line‐noise suppression at 50 Hz, stabilizing the effective analysis band and reducing predictable interference before feature extraction [179, 180]. Many pipelines also incorporate artifact detection and gating to prevent contaminated intervals from driving the decoder, followed by rejection, masking, or down‐weighting of affected epochs or channels during online operation [181, 182, 183]. Feature extraction is often selected to be inherently tolerant to residual contamination, with practical BCIs frequently relying on bandpower or envelope‐based summaries that provide stable control variables for motor‐intent decoding and state monitoring under nonstationary conditions [184, 185]. Together, these digital modules complement AFE conditioning and improve robustness to transient artifacts and session variability in practical BCIs.

## 4 Wireless Protocols in Scalable Systems

Advances in high‐resolution wired neural interfaces have driven a continuous increase in microelectrode array density, enabling the precise capture of fine‐scale cortical spatiotemporal dynamics [186, 187]. Recent high‐density µECoG technologies offer substantially improved spatial resolution and SNR compared with conventional µECoG electrode grids. These advancements allow for the accurate decoding of complex cortical functions [188], such as motor planning [189], language processing [190], and sensory representations [191].

While wired neural interfaces can readily support the transmission of such multi‐channel data at high sampling rates [192, 193, 194], they introduce fundamental limitations that restrict applicability in long‐term and freely behaving settings. Physical tethers and external connectors are vulnerable to mechanical instability, leading to motion‐induced artifacts and intermittent signal degradation [195]. Moreover, wired connections inherently constrain subject mobility and experimental paradigms, limiting their use in naturalistic behaviors and chronic recordings [196]. These challenges have motivated a growing shift toward wireless communication paradigms for BCI systems.

By eliminating physical tethers, wireless architecture improves robustness against motion artifacts and enhances subject mobility, enabling the scalable deployment of high‐channel‐count interfaces. Consequently, wireless communication schemes must be designed to accommodate increasing channel resolution and scalability, while operating under strict constraints on power consumption and device miniaturization. In particular, increasing spatial resolution directly drives higher data throughput requirements, necessitating high‐bandwidth communication protocols, whereas systems operating under tight power constraints often rely on lower‐throughput, energy‐efficient alternatives (Table 2).

**TABLE 2:** Wireless protocol overview for BCIs.

Protocol	Data rate	Power consumed	Range	Frequency band	Typical use case	References
Wi‐Fi	> 1 Mbps	300 – 800 mW	< 100 m	2.4/5/6 GHz	Local high‐throughput recording	[197, 198]
Bluetooth low energy	< 2 Mbps	1 – 100 mW	< 50 m	2.4 GHz	Wearable /implantable	[198, 199, 200]
Custom radio frequency	> 60 kBps	< 100 mW	< 0.5 m	Sub‐GHz/ GHz/ near‐field	Distributed/long‐term implants	[160, 201, 202]

### 4.1 Wi‐Fi Communication

Neural recording platforms with high channel counts and fine spatial resolution generate large streams of data, creating stringent requirements on communication bandwidth and scalability. As the number of recording channels increases, the aggregate data rate increases proportionally, making continuous transmission of raw or minimally processed neural signals increasingly challenging. In systems designed for high‐density EEG or large‐scale neural emulation, maintaining signal fidelity without heavy on‐device compression requires a communication link capable of supporting sustained, high‐throughput data streaming [203, 204, 205].

**FIGURE 5:**
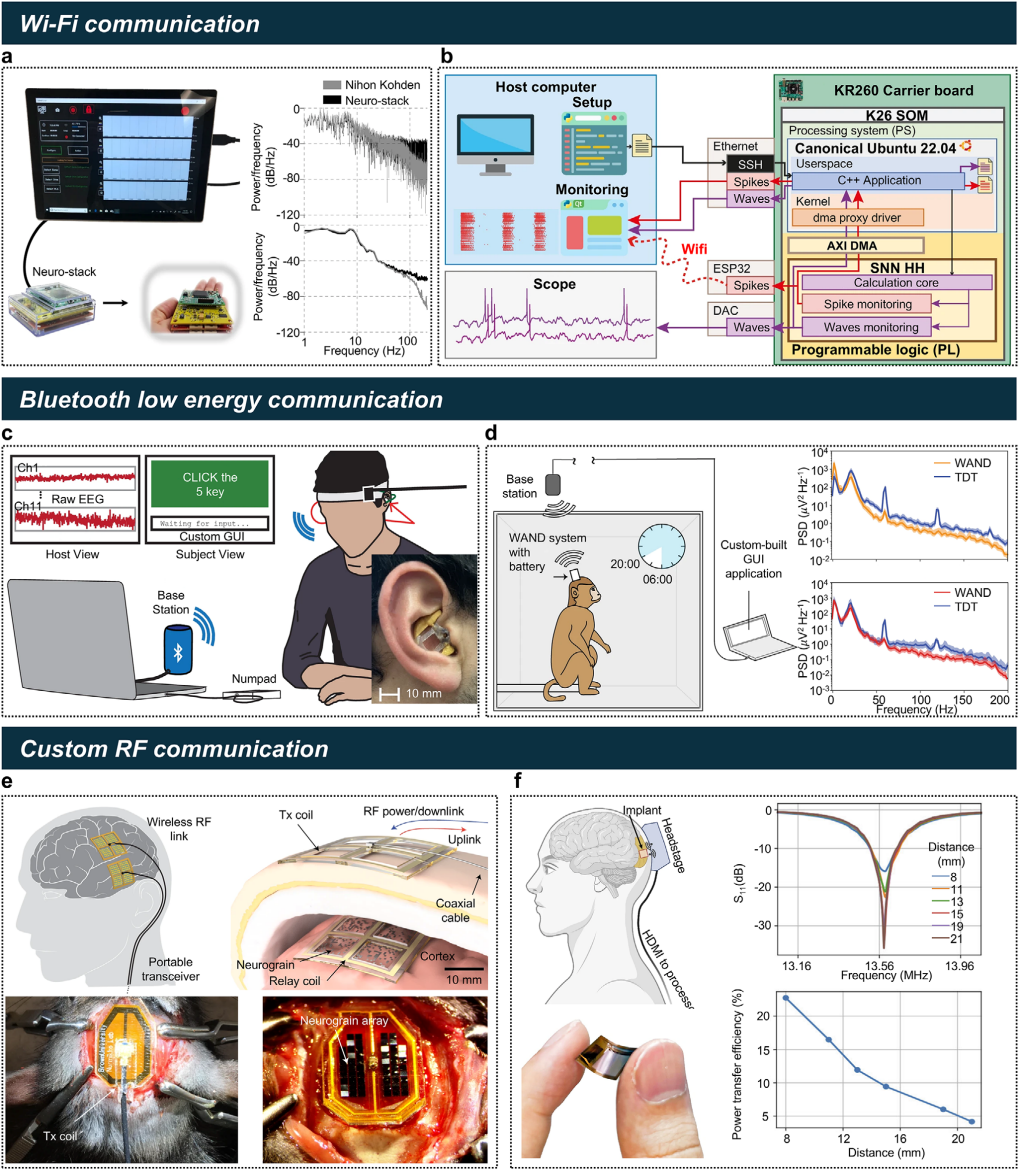
Scalable wireless communication architectures for high‐density neural recording. (A) The wireless Neuro‐stack hardware with its tablet GUI, and electrical validation results, demonstrating high signal fidelity comparable to wired clinical setups. Reproduced under terms of the CC‐BY license [209]. Copyright 2023, Springer Nature; (B) Schematic of the BiœmuS platform demonstrating wireless connectivity for a 1024 neuron system transmitting continuous membrane potentials. Reproduced under terms of the CC‐BY license [211]. Copyright 2024, Springer Nature; (C) BLE‐based wireless system for ear‐centered neural monitoring, displaying the system architecture and the miniaturized earbud device. Reproduced under terms of the CC‐BY license [222]. Copyright 2024, Springer Nature; (D) High‐throughput 96‐channel LFP streaming using the WAND system, showing the in vivo setup and signal validation. Reproduced with permission [223]. Copyright 2018, Springer Nature; (E) Scalable neurograin network, illustrating the system concept and in vivo demonstration of 48 distributed microimplants. Reproduced with permission [230]. Copyright 2021, Springer Nature; (F) Flexible 65 536‐electrode neural interface system for simultaneous 1024‐channel wireless recording. Reproduced with permission [231]. Copyright 2025, Springer Nature.

Wi‐Fi, based on the IEEE 802.11 family of wireless local area network standards, is well suited to meet these requirements, as its wide bandwidth and networking infrastructure enables reliable transmission of dense, multi‐channel neural data in real‐time [206, 207, 208]. For instance, Wi‐Fi based systems have demonstrated the ability to stream up to 128 channels of intracranial EEG while preserving signal quality comparable to wired clinical systems (Figure 5A) [209]. Additionally, the use of standard network protocols allows seamless integration with external devices, enabling precise synchronization through mechanisms such as the Network Time Protocol (NTP) [210]. Beyond electrode‐based acquisition, system‐level scaling further amplifies communication demands, as demonstrated by platforms such as BiœmuS (Figure 5B) [211]. This system emulates 1024 biophysically detailed Hodgkin‐Huxley neurons in real‐time and utilizes Wi‐Fi to stream not only spike events but also continuous membrane voltage waveforms, thereby imposing substantial bandwidth requirements for simultaneous data monitoring and closed‐loop interaction.

Despite these advantages, the employment of Wi‐Fi is fundamentally constrained by its high‐power consumption and associated thermal factors, limiting its applicability in fully implantable systems. As a result, Wi‐Fi is preferentially implemented in epidermal or other wearable neural interface systems, where device size, heat dissipation and power availability are less restrictive. In these skin‐mounted, semi‐implantable, or wearable configurations, Wi‐Fi facilitates continuous, high‐fidelity streaming of large‐scale neural data [212, 213, 214]. In contrast, fully implantable or highly compact systems that require long‐term autonomous operation under strict power budgets typically favor lower‐power communication protocols, even at the expense of reduced bandwidth or increased local data processing.

### 4.2 Bluetooth Low Energy

To address the power and size limitations of high‐bandwidth protocols like Wi‐Fi, there is increasing interest in Bluetooth Low Energy (BLE) for implantable and body‐area neural systems. In contrast to high‐throughput communication schemes, BLE is specifically suited for applications where strict power budgets, thermal safety, and device miniaturization are the dominant constraints, even if this limits achievable data rates and channel counts [200, 215]. Operating in the 2.4 GHz industrial, scientific, and medical (ISM) band, BLE enables energy‐efficient bidirectional communication with compact hardware implementations, making it well suited for anatomically constrained and chronic neural interfaces [216, 217, 218].

Building on these characteristics, recent BLE‐based neural interfaces have demonstrated reliable multi‐channel neural data transmission in miniaturized implantable or semi‐implantable form factors [219, 220, 221]. Specifically, ear‐centered and drowsiness‐monitoring platforms have successfully employed BLE links to sustain continuous real‐time operation (Figure 5C) [222]. By optimizing data packetization to maximize the throughput within the BLE bandwidth limit, these systems can reliably transmit approximately 11 channels of neural data. This capability strikes a critical balance between channel density and energy efficiency, enabling long‐term monitoring applications such as sleep staging and vigilance detection without the need for bulky batteries. In parallel, system‐level demonstrations have shown that BLE communication can be extended to substantially higher channel counts when required. For instance, the WAND system achieved real‐time streaming of up to 96 channels of uncompressed LFP over a BLE link by optimizing protocol parameters toward the 2 Mbps physical layer limit and selectively prioritizing signal types (Figure 5D) [223]. However, such configurations approach the upper limits of BLE throughput and often require protocol‐level customization, highlighting the inherent constraint between channel scalability and energy‐efficient communication. Overall, while BLE supports lower data rates compared to Wi‐Fi, it is preferentially adopted in implantable and body‐area systems where long‐term energy autonomy and compact device form factors are prioritized over maximum channel count and continuous high‐bandwidth streaming [200].

### 4.3 Custom Radio Frequency

As neural interface systems scale toward hundreds or thousands of recording and stimulation sites, the limitations of conventional wireless protocols such as Wi‐Fi and BLE become increasingly pronounced. Continuous streaming of broadband neural data imposes rapidly increasing data‐rate and power demands that are difficult to reconcile with miniaturized, chronically implantable hardware. In particular, the reliance of these protocols on onboard batteries or externally tethered power sources introduces constraints of device lifespan, size and long‐term biocompatibility [224, 225]. To address these challenges, custom RF communication schemes have emerged that co‐design wireless power transfer, node‐level addressing, and scheduled access to support large populations of distributed implants [168, 226, 227, 228, 229]. By enabling battery‐free operation through transcutaneous energy transfer, these systems eliminate the need for bulky energy storage components, making them especially well‐suited for fully implantable neural interfaces [227]. While these wireless protocols define the available communication bandwidth and power constraints, the efficiency of neural data transmission is also influenced by how the signals are represented and transmitted.

A representative implementation of this approach is the neurograin paradigm, in which sub‐millimeter microimplants communicate with an external hub via an approximately 1 GHz transcutaneous RF link and support individual device‐level addressing (Figure 5E) [230]. In vivo demonstrations have shown simultaneous operation of 48 neurograins distributed across the cortical surface, while system‐level analysis of the adopted time division multiple access (TDMA) protocol indicates potential scalability to several hundred nodes under the same communication framework. Related principles are effectively applied in monolithic high‐density neural interfaces, where external hardware orchestrates wireless power delivery and data aggregation to support massive channel scaling on a single substrate. A prominent example is a fully implantable, subdural brain–computer interface that integrates 65 536 electrodes onto a flexible chip (Figure 5F) [231]. By utilizing a wearable relay station to provide inductive power at 13.56 MHz and manage bidirectional impulse‐radio ultra‐wideband (IR‐UWB) data telemetry, this system achieves simultaneous wireless recording of 1024 channels. This architecture demonstrates how coordinating high‐bandwidth RF uplinks with near‐field power links enables the scaling of wireless recording density by orders of magnitude, supporting the decoding of fine‐grained cortical dynamics such as traveling waves in the visual cortex. These systems collectively demonstrate that battery‐free RF architectures, enabled by wireless power transfer and custom communication protocols, provide a scalable and biocompatible solution for high‐density invasive neural interfaces.

## 5 Temporal Resolution and Signal Fidelity

While different wireless protocols are employed for their respective specifications and applications, communication strategies serve a central role in shaping how effectively signals are transmitted and utilized in BCI systems. In this context, communication strategies operate at the signal level, shaping how temporal information is preserved or reduced to balance fidelity with bandwidth and power limitations [232]. Neural activity spans a wide range of timescales, from slow oscillations to millisecond‐level spiking events, necessitating communication strategies that can preserve temporal fidelity [233, 234, 235]. In general, reliable recording of action potentials require sampling rates on the order of tens of kilohertz, with ∼ 20 kHz widely regarded as a minimum for stable spike detection [236]. However, when combined with increasing channel density, such high temporal resolution leads to rapid growth in data throughput requirements, directly constrained by the wireless bandwidth and power limitations [237, 238, 239]. Such data‐intensive operation further imposes stringent constraints on bandwidth, power consumption, and latency in practical BCI implementations.

As a result, temporal resolution must be considered not only as a signal acquisition parameter but as a system‐level factor that directly influences the communication architecture. While protocol alone defines the raw broadband and payload available to these wireless systems, communication strategies determine how neural data are represented, prioritized, and transmitted within those constraints to balance temporal fidelity with bandwidth and power efficiency [240]. Contemporary BCI systems generally adopt one of two strategies: continuous streaming of raw neural signals to preserve full temporal detail, or event‐driven, feature‐based transmission to reduce communication load. This section examines these approaches and how they balance temporal fidelity with bandwidth efficiency.

### 5.1 Broadband Spike Streaming

Broadband spike streaming aims to preserve fast neural dynamics by continuously transmitting raw extracellular waveforms at sampling rates in the tens‐of‐kilohertz range, thereby maintaining precise spike timing and waveform morphology with minimal on‐device compression. Because data throughput in this paradigm scales linearly with both sampling frequency and channel count, sustaining tens‐of‐kilohertz sampling across multiple channels places stringent demands on the recording front‐end, power delivery, packaging, and wireless telemetry. As a result, recent systems adopting broadband streaming have increasingly relied on co‐optimization of AFE design, mechanical integration, and communication links to support stable high‐rate acquisition under freely behaving conditions [159, 241, 242, 243].

**FIGURE 6:**
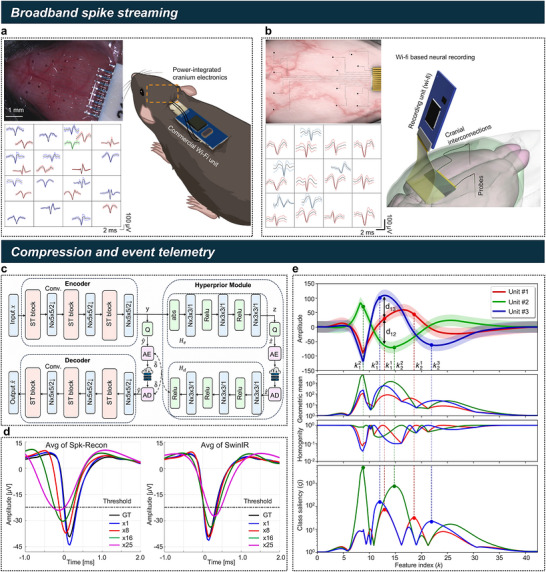
Wireless transmission strategies for preserving temporal fidelity. (A) Power‐integrated cranial electronics with commercial Wi‐Fi, enabling wireless single‐unit recordings at ∼20 kS/s from distributed brain regions. Reproduced with permission [241]. Copyright 2024, The American Association for the Advancement of Science; (B) Long‐range Wi‐Fi streaming architecture with conformal cranial interconnects, supporting stable multi‐site spike acquisition over meter‐scale distances. Reproduced under terms of the CC‐BY license [213]. Copyright 2024, Springer Nature; (C) Overview of a neural signal compression framework in which multi‐channel inputs are encoded into a quantized latent representation, transmitted with auxiliary side information, and reconstructed via a decoder, enabling reduced communication bandwidth while preserving original signal structure. Reproduced with permission [248]. Copyright 2026, Elsevier; (D) Spk‐Recon framework, demonstrating the accurate reconstruction of high‐frequency spike waveforms from reduced‐bandwidth representations. Reproduced under terms of the CC‐BY license [249]. Copyright 2024, Springer Nature; (E) On‐implant spike sorting for event‐level telemetry, depicting the optimization of discrimination points to maximize class separability for online processing. Reproduced under terms of the CC‐BY license [250]. Copyright 2020, Springer Nature.

Representative implementations illustrate how different wireless strategies extend broadband streaming beyond tethered setups. Power‐integrated cranial electronics that combine energy storage, interconnect routing, and compact Wi‐Fi telemetry modules reduce mechanical burden while sustaining wireless single‐unit recordings at ∼ 20 kS/s from probes distributed across multiple brain regions (Figure 6A) [241]. Building on such system‐level integration, long‐range Wi‐Fi‐based streaming architectures integrated with conformal cranial interconnects have further demonstrated stable single‐unit recordings at sampling rates of ∼ 20 kS/s while maintaining meter‐scale operating distances and supporting simultaneous multi‐site spike acquisition (Figure 6B) [213]. As spatial coverage increases further, the wireless uplink itself becomes the limiting factor for sustaining broadband sampling rates across large populations of neurons. To address this bottleneck, custom wideband RF solutions such as IR‐UWB have been developed to maximize data throughput. A fully integrated IR‐UWB transmitter exemplifies this capability, achieving a data rate of 1.66 Gbps through a power‐efficient 3D hybrid modulation scheme. This extreme bandwidth enables the transmission of raw, minimally processed neural signals from over 1000 channels simultaneously. Critically, such high‐throughput streaming preserves the full temporal fidelity of neuronal waveforms, allowing computationally intensive tasks to be offloaded to powerful external processors rather than being constrained by the limited power budget [244]. Together, these systems highlight how broadband spike streaming prioritizes temporal fidelity by shifting complexity to communication and system integration, rather than local data reduction.

### 5.2 Signal Compression and Event‐Driven Telemetry

While broadband spike streaming preserves temporal fidelity by continuously transmitting high‐rate raw waveforms, its data rate and power requirements scale rapidly with sampling frequency and channel count, making sustained operation challenging under constrained wireless power budgets. Feature‐only and event‐driven telemetry addresses this limitation by shifting part of the temporal information handling on‐device and transmitting compact representations that preserve task‐relevant timing cues with far lower communication load [245, 246, 247]. Accordingly, feature‐only and event‐driven telemetry prioritizes compact representations of temporally informative activity over continuous broadband waveform transmission.

Representative implementations illustrate this progression from continuous‐signal compression to reconstruction‐enabled temporal utility and event‐level telemetry. An autoencoder‐based compression framework encodes continuous multi‐channel neural signals into a low‐dimensional latent representation for transmission and reconstructs the signals externally, directly targeting the data‐rate and power bottlenecks associated with raw streaming (Figure 6C) [248]. Notably, quantitative analysis demonstrates that this compression scheme enables an 18‐fold increase in channel scalability under a fixed implantable power budget. Building on this idea, Spk‐Recon demonstrates that transmitting a reduced‐bandwidth neural representation can still support spike‐centric analyses (Figure 6D) [249]. Crucially, it enables downstream spike detection without continuous broadband telemetry by reconstructing high‐frequency structures that maintain high morphological similarity to ground truth waveforms. At the most communication‐efficient extreme, on‐implant spike sorting replaces waveform transmission with event‐level outputs, where the implant performs online sorting to produce spike labels/events for telemetry (Figure 6E) [250]. An external module performs offline clustering and parameter refinement to calibrate the on‐implant sorter, specifically by identifying optimal discrimination points that maximize class separability based on geometric distance and homogeneity. This approach minimizes wireless payload while retaining temporally informative spike timing for decoding and closed‐loop use.

When comparing the two approaches, their use is strongly application‐dependent and reflects a fundamental trade‐off between temporal fidelity and communication efficiency. Broadband spike streaming preserves continuous raw waveforms, enabling precise spike timing, waveform morphology analysis, and flexible post hoc decoding. As a result, it is typically favored in applications that require high‐resolution neural mapping, offline analysis, or advanced decoding algorithms, where access to full signal detail allows for improved spike sorting accuracy, feature extraction, and model generalization [251]. In contrast, compressed or event‐driven strategies reduce communication load by transmitting compact representations or discrete neural events, which significantly lowers power consumption and enables long‐term operation in resource‐constrained or implantable systems [252]. However, this reduction in data fidelity can lead to loss of fine temporal structure and waveform information, limiting the ability to perform detailed spike sorting or recover subtle neural dynamics. In practice, this may degrade decoding performance in tasks that rely on precise spike timing or high‐dimensional signal features and may constrain the use of more complex or adaptive decoding algorithms. Despite these limitations, such approaches remain effective in real‐time closed‐loop systems where task‐relevant features can be extracted reliably, and where reduced latency and power consumption are prioritized over maximum signal fidelity.

## 6 AI Decoding and Closed‐Loop Inference

AI decoding in modern BCIs has shifted from offline analysis toward real‐time inference that can drive closed‐loop feedback, where reliability and response time are as critical as accuracy [253, 254, 255]. In this chapter, we organize decoding and closed‐loop designs around four input representations: continuous neural waveforms, graph‐structured representations that encode inter‐channel relationships, learned compressed or latent codes, and spike‐derived event or count streams. We next review the evolution of decoding paradigms from threshold and handcrafted feature‐based approaches to machine learning and deep‐learning decoders and then summarize resource‐aware and on‐device strategies that enable practical low‐latency closed‐loop operation.

### 6.1 Threshold‐Based Decoding

**FIGURE 7:**
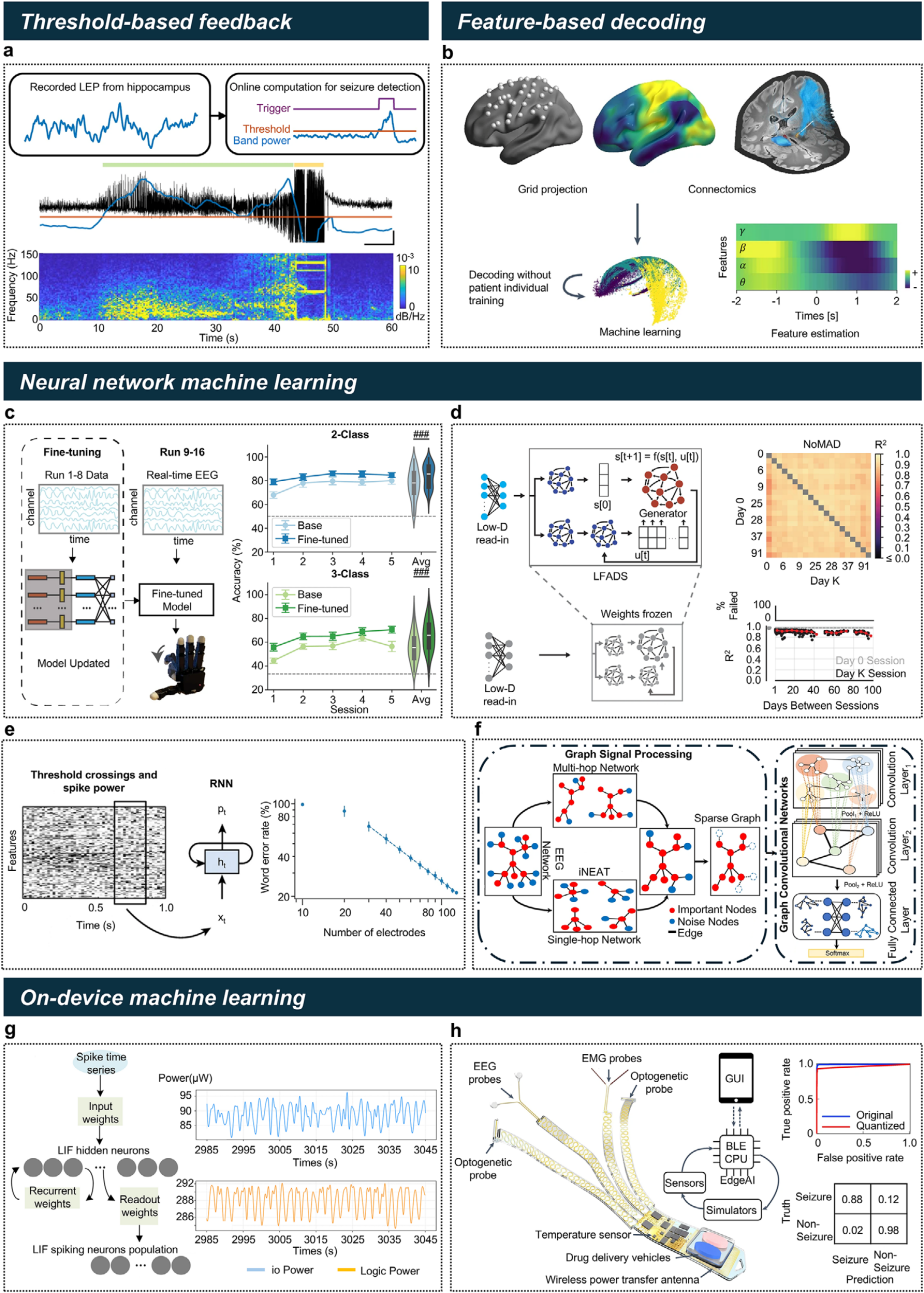
AI decoding paradigms for closed‐loop neural interfaces. (A) Threshold‐based closed‐loop stimulation, triggering responsive feedback when real‐time LFP band power exceeds a preset seizure threshold. Reproduced under terms of the CC‐BY license [258]. Copyright 2022, Springer Nature; (B) Connectomics‐based decoding pipeline mapping electrodes to brain networks, extracting features, and enabling decoding without patient‐specific training. Reproduced with permission [269]. Copyright 2025, Springer Nature; (C) Continuous EEG decoding with session‐specific fine‐tuning, enabling precise online control of a multi‐finger robotic hand. Reproduced under terms of the CC‐BY license [184]. Copyright 2025, Springer Nature; (D) Latent Factor Analysis via Dynamical Systems (LFADS) framework for stable, low‐dimensional decoding. Reproduced with permission [285]. Copyright 2025, Springer Nature; (E) RNN‐based spike event decoding for bandwidth‐efficient telemetry. Reproduced under terms of the CC‐BY license [42]. Copyright 2024, Springer Nature; (F) Graph‐structured decoding network for modeling inter‐channel dependencies and spatial priors. Reproduced under terms of the CC‐BY license [292]. Copyright 2023, Springer Nature; (G) On‐chip spiking neural network for real‐time epilepsy detection under sub‐milliwatt power. Reproduced with permission [302]. Copyright 2024, Elsevier; (H) Wearable edge inference system using quantized models for low‐latency seizure monitoring. Reproduced with permission [306]. Copyright 2023, Springer Nature.

Threshold‐based closed‐loop control has been extensively investigated and translated into clinical systems. In this approach, systems compute low‐complexity features in real time, including band power, line length, and area under the curve, and trigger stimulation or feedback when individualized thresholds indicate a transition in neural state, such as seizure onset or increased pathological beta activity [256, 257]. A representative implementation demonstrates this mechanism by monitoring the band power of LFP in real time. When the integrated power exceeds a preset seizure threshold, the system immediately triggers sequential narrow‐field stimulation to terminate the event (Figure 7A) [258]. In drug‐resistant epilepsy, the responsive neurostimulation (RNS) system exemplifies this paradigm by combining configurable bandpass, line‐length, and area detectors to identify events and deliver responsive stimulation [259, 260]. In Parkinson's disease, adaptive DBS commonly uses subthalamic LFP beta‐band power as a control signal. Systems typically define patient‐specific beta bands and, in some implementations, center the band on the individual beta peak, to switch stimulation on and off or to adjust amplitude using a dual‐threshold policy, improving efficiency while limiting side effects [261, 262, 263]. However, fixed thresholds offer limited expressivity and often require repeated calibration as signal statistics drift with behavior, electrode conditions, and disease state. These limitations have driven a shift toward handcrafted feature‐based decoding pipelines and, subsequently, machine‐learning and deep‐learning approaches.

### 6.2 Handcrafted Feature‐Based Decoding

Handcrafted feature‐based decoding pipelines extend simple threshold rules by explicitly engineering informative descriptors of neural activity and training classical machine‐learning models to separate states. Typical features span time‐domain morphology, spectral band power and band ratios, time–frequency energies, multichannel spatial statistics and inter‐channel relationship measures such as coherence or phase synchrony. These features are then classified using standard linear and tree‐based models, including logistic regression, linear discriminant analysis (LDA), support vector machines (SVM), decision trees, random forests, and gradient‐boosted trees [264, 265, 266, 267, 268]. Notably, the same robust feature engineering underlying this classification has also enabled calibration‐free decoding, allowing models to generalize across patients without requiring individual retraining (Figure 7B) [269]. To identify the specific neural descriptors driving such generalization and support model validation, post hoc interpretability tools, including SHapley Additive exPlanations (SHAP) and permutation‐based importance, are used to quantify feature contributions and support feature selection and model validation [270, 271, 272]. Despite these advances and strong performance in low‐data settings, constructing such pipelines often requires substantial domain‐specific tuning, while also scaling poorly as channel count and feature dimensionality grow, motivating deep‐learning approaches that learn representations directly from raw or minimally processed signals.

### 6.3 Deep Learning‐Based Decoding

To support neural decoding under practical system constraints, a range of input representations have been developed that differ in how neural signals are preserved, transformed, or compressed. These approaches span from continuous waveform and time‐frequency representations that retain detailed signal information, to more compact representations such as latent codes and spike‐based event streams that reduce communication and computational demands. In parallel, graph‐based methods focus on capturing inter‐channel relationships to improve robustness and decoding stability.

For systems that emphasize raw signal fidelity, minimally processed continuous waveforms are directly input into neural networks to learn and analyze the temporal context and spatial patterns jointly [273, 274, 275]. For scalp EEG applications, compact architectures such as EEGNet provide a widely used baseline that features strong performance with limited parameters and training data [276]. This method is also conventional for real‐time control as no manual feature extraction is needed.

Many studies also employ fixed time‐frequency representations, transforming continuous EEG into short‐time Fourier transform (STFT)‐based spectrograms and implementing convolution neural networks (CNN) or Transformer models. Similar to the continuous waveforms, this approach enables joint learning of temporal and spatial features, while improving signal separability by explicitly representing frequency‐domain characteristics [277, 278, 279].

When deployed in closed‐loop systems, these continuous‐input decoding approaches directly couple real‐time inference with feedback, making latency and stability critical alongside decoding performance. For long‐term monitoring systems, addressing neural non‐stationarity is essential to maintain stable and reliable decoding performance as signal characteristics change over time [280]. To maintain this stability against neural non‐stationarity, recent frameworks utilize session‐specific fine‐tuning of pre‐trained deep learning models, enabling precise and adaptive online control of complex devices such as multi‐finger robotic hands (Figure 7C) [184]. Similar continuous‐input decoding appears in higher‐level neuroprosthetic applications, where multichannel cortical activity is mapped to structured outputs such as text or speech, illustrating how end‐to‐end models can connect continuous neural dynamics to downstream communication interfaces [281]. While these methods aim to preserve full signal fidelity and maintain stability over long‐term operation, continuous multi‐channel streaming imposes substantial communication demands that can exceed available bandwidth and power constraints [240].

For balancing efficiency while maintaining the relative fidelity of the original raw signal, low‐dimensional representations, where models operate on compact latent codes, provide a means of compressing neural activity into task‐relevant features that reduce computational overhead while preserving essential temporal and spatial dynamics for downstream decoding [282, 283, 284]. Latent dynamical models such as Latent Factor Analysis via Dynamical Systems (LFADS) represent population activity through a low‐dimensional generator state, providing a learned code that can support decoding while reducing bandwidth and facilitating more stable downstream inference (Figure 7D) [285]. In this view, the core design choice is not only what to decode, but what representation to communicate, so that the loss of information induced by compression is managed by learning rather than by manual feature selection.

At the most communication‐efficient extreme, decoders operate on spike‐derived event or count streams rather than raw waveforms. Spike trains are typically represented as time‐stamped events or binned count/rate sequences and encoded using sequence models such as recurrent neural networks (RNNs), temporal convolutions, or Transformer attention to capture timing structure and longer‐range dependencies [286, 287, 288]. In this setting, deep models treat spike trains as the primary input and map event timing and rates into compact embeddings for classification or state decoding. This approach significantly reduces telemetry bandwidth while retaining temporally informative patterns, thereby confirming the critical role of high‐density arrays in enhancing communication (Figure 7E) [42]. This event‐based representation reduces telemetry while retaining temporally informative spiking patterns that can drive closed‐loop decisions.

While the preceding approaches focus on improving communication efficiency through more compact signal representations, an alternative direction emphasizes improving decoding robustness by explicitly modeling relationships across channels [289, 290, 291]. Graph‐structured formulations build an input representation around channel‐to‐channel dependencies and apply message passing or graph convolution to learn network‐level biomarkers and spatial priors, which is especially useful when electrode geometry or functional coupling carries meaningful structure (Figure 7F) [292].

While these decoding approaches span a wide range of input representations and offer improvements in performance, constraints remain extant across all categories when considered for practical closed‐loop deployment. Continuous waveform‐based decoding, although preserving full signal fidelity, imposes substantial data throughput and computational demands that can introduce latency and limit real‐time responsiveness in feedback‐driven systems [293]. Time–frequency‐based methods, such as STFT spectrogram representations, improve signal separability but incur additional preprocessing overhead and remain sensitive to temporal variability and non‐stationarity [294]. Latent representation approaches, including models such as LFADS, reduce dimensionality and communication burden but rely on learned priors that may not generalize robustly across sessions or subjects without retraining [295]. At the most communication‐efficient extreme, spike‐based or event‐driven representations significantly reduce telemetry bandwidth but are highly susceptible to noise, which can limit overall performance and applicability [296]. Graph‐based decoding frameworks improve robustness by modeling inter‐channel dependencies; however, they introduce additional computational complexity and require accurate modeling of spatial relationships, which may vary across recording conditions [297]. Across all approaches, neural non‐stationarity remains a fundamental challenge, as signal characteristics shift over time and across users, necessitating recalibration or adaptive learning strategies to maintain stable performance [285]. These limitations highlight that trade‐offs between fidelity, efficiency, robustness, and generalization persist, underscoring the significance of adaptive and resource‐efficient decoding strategies for reliable closed‐loop BCI deployment.

### 6.4 On‐Device Processing and Edge Inference

Before on‐device deep inference became practical, many closed‐loop devices relied on simple on‐node detectors that compute lightweight biomarkers and trigger stimulation when individualized thresholds are exceeded [261, 298]. More recently, neural inference has moved onto the device, enabling continuous decoding while transmitting only decisions or sparse markers instead of high‐rate waveforms [299, 300, 301]. For example, an epilepsy‐detection system deploys a trained spiking neural network on a neuromorphic edge processor for on‐chip, real‐time inference under sub‐milliwatt power budgets, supporting rapid detection without sustained broadband telemetry (Figure 7G) [302].

Beyond neuromorphic accelerators, an increasing number of wearable and implantable prototypes implement lightweight neural networks directly on low‐power microcontrollers, co‐locating sensing, preprocessing, inference, and wireless communication within a compact node [303, 304, 305]. In these systems, designers typically use quantized or sparsified models and fixed‐point kernels to fit tight memory and compute budgets, so the device can issue low‐latency state estimates that drive stimulation or haptic feedback while keeping radios mostly idle (Figure 7H) [306]. This integration makes the form factor and energy profile compatible with long‐term wearable operation, and it enables practical closed‐loop deployment by turning continuous biosignals into locally decoded control variables rather than continuously streamed data.

## 7 Applications for BCI Systems

Recent research in BCIs has expanded beyond laboratory‐based proof‐of‐concept demonstrations toward applications in clinical rehabilitation [307, 308], assistive technologies for daily living [309], cognitive state monitoring [20], and interactive or creative use cases [310]. This expansion has been driven by sustained advances in electrode design and system architecture, particularly improvements in electrochemical performance and the development of environment‐adaptive electrode interfaces. When combined with machine learning‐ and deep learning‐based decoders capable of extracting complex spatiotemporal features from multichannel neural signals, these advances enable closed‐loop interactions that provide real‐time sensory feedback or adaptive interventions [255, 311].

Although substantial advances have been achieved, additional challenges emerge when transitioning from laboratory demonstrations to clinical or everyday environments. At the application level, maintaining signal stability under repeated use, ensuring sufficient system responsiveness for real‐time interaction, and supporting long‐term adaptability remain important considerations [237, 312, 313]. To enable a consistent and critical comparison across studies, we explicitly evaluate BCI systems based on three key criteria: (i) signal stability, defined as the ability to maintain reliable neural signal quality over time, including resistance to impedance drift and signal degradation; (ii) real‐time responsiveness, referring to the latency and continuity of closed‐loop interaction between neural activity and system output; and (iii) long‐term usability, encompassing chronic operation, energy efficiency, and user adaptability in practical environments. Accordingly, this section analyzes BCI applications from the perspective of sustained and reliable operation in real‐world settings. Tables summarize representative human‐subject demonstrations and highlight key application‐level parameters, including task design, neural signal modality, decoding strategy, and system latency, which are further interpreted in relation to these three evaluation criteria. Building on this framework, the following subsections examine representative studies within each application category to assess how these requirements are addressed in practice.

### 7.1 Sensory Restoration and Substitution

Sensory restoration and substitution BCIs aim to reconstruct or replace lost sensory functions by decoding neural activity and delivering artificial stimuli that can be perceived by the user. These approaches emphasize the formation of stable stimulus–percept mappings through closed‐loop neural interfaces, enabling the recovery of perception such as touch, vision, audition, and speech. Early sensory restoration and substitution systems primarily aimed to demonstrate that sensory information could be functionally replaced by decoding neural activity and converting it into visual or mechanical feedback [314, 315]. While these approaches established the feasibility of sensory signal encoding, they are limited in supporting the continuity and consistency required for stable perception. In particular, rigid or semi‐rigid electrodes and tethered experimental setups are highly susceptible to micromotion and changes in contact conditions, often leading to perceptual distortions and degraded reliability of feedback [63]. Such limitations not only reduce signal quality but also hinder stable stimulus–percept mapping, constraining user adaptation and long‐term use.

Recent work in sensory restoration and substitution has therefore moved beyond isolated decoding performance toward validating personalization, reliability, and long‐term usability through human‐subject demonstrations (Table 3). For instance, studies involving individuals with SCI show that sub‐perceptual sensory signals evoked by peripheral stimulation can be decoded in real time to restore both tactile perception and motor function [315]. Using SVM‐based decoder with closed‐loop sensory feedback, object contact detection accuracy exceeding 90% are achieved, enabling simultaneous grip force modulation and motor control. Notably, the stability of the neural interface over more than 5 years directly demonstrates the long‐term viability of sensory restoration BCIs in human users. These results highlight the importance of maintaining stable, bidirectional sensorimotor integration in real‐world conditions, where consistent interface performance over time becomes a primary design requirement.

**TABLE 3:** Applications of BCIs in sensory restoration and raehabilitation.

Task	Signal modality		Performance	Latency	Decoding algorithm	Reference
	Recording	Stimulation				
Brain‐to‐voice prosthesis to restore communication	ECoG	Voice synthesis	WER^a)^ 25.5%	< 10 ms	RNN	[281]
Restoration of touch	Utah MEA	Skin electrical stimulation	ACC^b)^ 92%–95% (touch detection)	2.9 s	SVM	[315]
Brain‐to‐voice prosthesis to restore communication	ECoG, MEA, EMG	Voice synthesis	PER^c)^ 38.7%	11.83 ms	RNN	[316]
Restoration of motor control	Intracortical signal	Epidural electrical stimulation	ACC 91.5% (arm kinematic during pull)	15 ms	Threshold strategy	[317]
Restoration of tactile sensation	EMG	Nerve electrical stimulation	ACC 99.3% (5‐class)	< 50 ms (detection of slippage), ∼ 500 ms (overall control)	Nonlinear logistic regression algorithm	[318]
Closed‐loop deep brain stimulation	ECoG	Deep brain stimulation	ACC 92.55% (detection of movement execution)	N/A^d)^	LDA	[319]
Restoration of motor control	ECoG	Epidural electrical stimulation	ACC 74% (7‐class)	1.1 s	Aksenova/Markov‐switching multilinear algorithm	[320]
Brain‐to‐voice prosthesis to restore communication	Utah MEA	Voice synthesis	PER 37.1%	< 10 ms	Transformer‐based model	[321]

^a^ WER, word error rate;

^b^ ACC, accuracy;

^c^ PER, phoneme error rate;

^d^ N/A, not applicable

**FIGURE 8:**
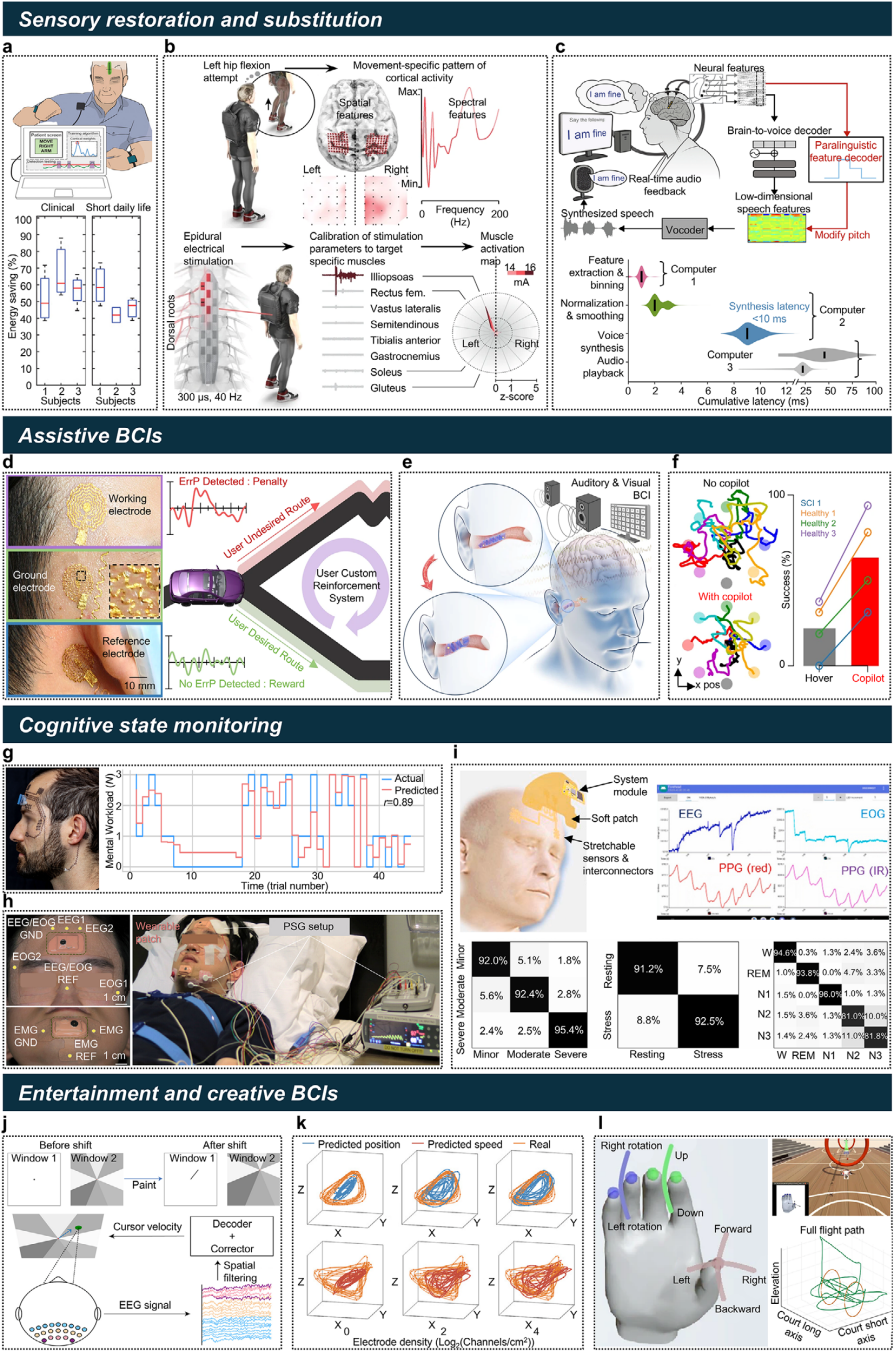
Real‐world applications of BCI systems. (A) Adaptive deep brain stimulation system delivering closed‐loop stimulation for tremor suppression. Reproduced with permission [319]. Copyright 2020, The American Association for the Advancement of Science; (B) Brain–spine interface linking wireless cortical recordings to epidural spinal cord stimulation for gait restoration. Reproduced under terms of the CC‐BY license [320]. Copyright 2023, Springer Nature; (C) Brain‐to‐voice neuroprosthesis decoding intracortical signals for real‐time speech synthesis in individuals with ALS. Reproduced with permission [321]. Copyright 2025, Springer Nature; (D) Assistive mobility system incorporating EEG‐based detection of error‐related potentials for implicit feedback and closed‐loop correction. Reproduced under terms of the CC‐BY license [328]. Copyright 2022, Springer Nature; (E) In‐ear EEG–based assistive BCI enabling spelling and auditory attention decoding with a minimal number of electrodes. Reproduced under terms of the CC‐BY license [330]. Copyright 2023, Springer Nature; (F) Shared‐autonomy assistive BCI combining noninvasive EEG with AI‐driven control for cursor and robotic arm operation. Reproduced with permission [329]. Copyright 2025, Springer Nature; (G) Ultrathin electronic tattoo–based cognitive BCI for continuous monitoring of mental workload using frontal electrophysiological signals. Reproduced with permission [337]. Copyright 2025, Elsevier; (H) Wireless soft patch–based system integrating EEG, EOG, and EMG for long‐term sleep monitoring in home environments. Reproduced with permission [338]. Copyright 2023, The American Association for the Advancement of Science; (I) Wireless, soft, forehead‐mounted multimodal biopatch enabling unified cognitive state monitoring through integrated physiological sensing. Reproduced with permission [339]. Copyright 2025, Elsevier; (J) Visual tracking BCI translating EEG correlation patterns into continuous cursor or object motion for interactive drawing and gaming tasks. Reproduced with permission [347]. Copyright 2024, Elsevier; (K) High‐density µECoG–based BCI enabling real‐time decoding of three‐dimensional movement for game control. Reproduced under terms of the CC‐BY license [348]. Copyright 2025, John Wiley and Sons; (L) Intracortical BCI supporting continuous decoding of finger‐level motor intentions for multi‐degree‐of‐freedom interaction in virtual environments. Reproduced under terms of the CC‐BY license [274]. Copyright 2025, Springer Nature.

In a clinical rehabilitation context, a fully implantable closed‐loop DBS system is applied to patients with essential tremors, delivering stimulation only when movement‐related neural signatures are detected (Figure 8A) [319]. Over 6 months, the system shows stable signal performance, with no significant deterioration in cortical features and only moderate impedance variation (∼ 12.35% per month). In terms of real‐time responsiveness, the average delay from movement onset to peak therapeutic stimulation is 1.72 s, indicating adaptive but not fully instantaneous control. For long‐term usability, the system reduces energy consumption by approximately 58% compared with conventional open‐loop DBS, supporting its practicality for chronic clinical use.

Sensorimotor restoration further encompasses gait recovery. In brain–spine interface studies, wireless ECoG signals from the motor cortex are linked in real time to epidural spinal cord stimulation, enabling near‐natural walking behavior (Figure 8B) [320]. Over nearly 1 year of use, the system maintains stable signal quality and decoding accuracy during walking, demonstrating robust signal stability. The continuous coupling between cortical activity and spinal stimulation further supports real‐time responsiveness for adaptive motor control. Importantly, partial voluntary gait function persists even after the system is deactivated, indicating durable functional integration beyond assistive operation and supporting long‐term usability.

Alongside cortical‐spinal coupling, neural interface applications have increasingly extended to the spinal cord and peripheral nervous system, where direct modulation of motor and sensory pathways enables complementary restoration strategies. An electronic dura mater integrating electrical stimulation with localized drug delivery was applied to support long‐term neuromodulation and rehabilitation following paralysis [322]. The platform maintained stable electrode impedance over more than 5 weeks of chronic implantation in vivo, while the colocalized electrical and chemical stimulation enabled paralyzed rats to recover walking ability after 3 weeks of implantation. However, because this approach primarily focuses on sustained neuromodulation rather than rapid closed‐loop interaction, real‐time responsiveness is not explicitly addressed. In terms of long‐term usability, the observed functional recovery suggests therapeutic potential, although extended chronic validation remains limited. In parallel, adaptive peripheral nerve interfaces have further advanced rehabilitation‐oriented neuromodulation by enabling conformal and stable coupling to target nerves via self‐closing or self‐wrapping architectures, supporting functional rehabilitation through targeted activation of peripheral motor pathways and restoration of sensory feedback [323, 324]. Together, these approaches demonstrate how closed‐loop neural interfaces can restore motor function through distributed modulation across brain, spinal, and peripheral pathways, while also establishing a foundation for extending restoration toward more complex sensory and cognitive modalities.

Sensory restoration extends to higher‐order modalities such as speech and audition. In a “brain‐to‐voice” neuroprosthesis for individuals with ALS, intracortical signals are decoded to synthesize speech in real time with sub‐10 ms latency, enabling immediate auditory feedback during attempted speaking (Figure 8C) [321]. This system supports a causal, real‐time auditory feedback loop, allowing users to immediately perceive the synthesized speech and use it to guide ongoing vocal intent, restoring a functional perception–action loop for communication. Deep learning‐based decoding captures not only phonemic content but also paralinguistic features, such as intended speaking rate and prosody, by exploiting precentral gyrus activity that jointly encodes content and expression. In addition, spurious synthesis is effectively suppressed during non‐speech conditions, including noisy vocalizations, coughing, and surrounding conversations, indicating robust decoding specificity in practical settings. However, long‐term usability remains to be further validated, particularly in terms of chronic implantation, sustained user adaptation, and reliable performance during prolonged everyday communication. These results demonstrate the restoration of speaker identity and auditory‐guided expressive communication, supporting reliable and sustained use in real‐world human settings.

### 7.2 Assistive Technology in BCIs

In contrast to sensory restoration approaches that aim to reconstruct perception, assistive BCIs translate neural signals into external actions or communication outputs. Assistive and communication BCIs have evolved toward portable systems that validate real‐time operation, signal stability, and long‐term usability under everyday conditions. Recent studies emphasize wearable form factors and adaptive decoding or control strategies that accommodate inter‐user variability and changing contexts, extending BCIs from short‐term demonstrations to continuously usable assistive technologies (Table 4). For example, skin‐conformal EEG systems integrating ultrathin nanomembrane electrodes with flexible circuits enable stable long‐term signal acquisition without gels or rigid caps while supporting fully wireless architectures [325]. Time‐domain CNN‐based universal classifiers further enhance robustness to inter‐subject EEG variability, supporting reliable and continuous real‐time communication and mobility assistance. While these systems demonstrate robust performance in controlled settings, evidence for sustained stability and usability under real‐world operation remains to be further established.

**TABLE 4:** Applications of BCIs in assistive technologies.

Task	Signal modality		Performance	Latency	Decoding algorithm	Reference
	Recording	Stimulation				
Robotic finger control	EEG	N/A	ACC 80.56% (2‐class)	1 s	DNN^a)^	[184]
Visual stimulus decoding	ECoG	N/A	ACC 97.8% (5‐class)	200 ms	CNN	[231]
Attempted handwriting	Intracortical signal	N/A	WER 1.5%	0.4 s	RNN + language model	[253]
Control of home appliance	EEG, EOG	N/A	ACC 92.80% (4‐class)	N/A	EMSI^b)^	[326]
Control of machine (wheelchair, vehicle, presentation)	EEG	N/A	ACC 94.54% (5‐class)	N/A	CNN	[325]
Assistive mobility with cognitive monitoring	EEG	N/A	ACC 74.26%	8.4 s	Logistic regression model	[327]
Autonomous machine decision‐making	EEG	Emergency interruption, reinforcement	ACC 98.57% (5 trials)	0.05–0.35 s input window	DNN + LSTM^c)^	[328]
Control of machine (computer cursor, robotic arm)	EEG	N/A	N/A	N/A	CNN + Kalman filter	[329]

^a^ DNN, deep neural network;

^b^ EMSI, multivariate synchronization index;

^c^ LSTM, long short‐term memory

Beyond command decoding, adaptive assistive BCIs increasingly incorporate user‐state awareness. EEG‐derived affective or stress‐related features have been integrated into mobility systems to dynamically adjust control modes, improving safety during prolonged use [327]. These approaches require robust performance under practical everyday conditions, such as environmental noise and user fatigue, together with sustained long‐term usability. Related approaches employ ear‐centered EEG to detect error‐related potentials (ErrP) as implicit feedback, enabling closed‐loop correction of autonomous system decisions and continuous supervision in real‐world navigation tasks (Figure 8D) [328]. Tattoo‐like dry electrodes in an earbud‐like form factor reduce artifacts and enable reliable ErrP recording during walking and driving. This gel‐free, lightweight design enables real‐time AI correction and supports prolonged wear. Further advances in wearable miniaturization broaden assistive BCI applicability. In‐ear EEG devices maintain stable electrode contact through a self‐supporting structure, enabling real‐time SSVEP‐based spelling and auditory attention decoding during everyday conversation with a minimal number of electrodes (Figure 8E) [330]. This design supports long‐term wear while reducing calibration burden.

At the system level, shared autonomy architectures combining noninvasive EEG with AI‐driven controllers mitigate low SNR and signal variability by delegating fine‐grained control to AI while preserving user intent (Figure 8F) [329]. Closed‐loop evaluations across three healthy participants and one participant with SCI demonstrate improved cursor and robotic arm control performance. In particular, the AI co‐pilot integrates neural signals with environmental context in real time, accelerating task completion by up to 4.3‐fold and reducing fine control time. This shared autonomy framework also reduces user effort, suggesting suitability for longer sessions and potential for practical deployment. Recent advances in assistive BCIs demonstrate meaningful progress toward practical deployment by improving real‐time responsiveness, signal robustness, and wearable usability across diverse application scenarios. At the same time, future work should extend current short‐term evaluations to establish long‐term robustness and usability in real‐world settings.

### 7.3 Cognitive State Monitoring

BCI‐based cognitive state monitoring has expanded toward real‐time estimation of higher‐order cognitive states (e.g., attention, drowsiness, mental workload, and executive function) and their direct integration into safety management, performance optimization, and cognitive rehabilitation [331, 332, 333]. Beyond improvements in decoding accuracy, recent studies increasingly emphasize sensor placement and form‐factor optimization to ensure signal stability, wearability, and reliability during prolonged use in real‐world environments [334] (Table 5). Compared with sensory restoration and assistive BCIs, cognitive state monitoring places greater emphasis on continuous operation, long‐term wearability, and user comfort in everyday environments, rather than on immediate responsiveness alone. Wearable platforms positioned around the ear, integrated into headphones, or mounted on the face demonstrate continuous monitoring over tens of hours with robust cognitive state classification, supporting the feasibility of sustained everyday cognitive monitoring [222, 331].

**TABLE 5:** Applications of BCIs for cognitive monitoring.

Task	Signal modality		Performance	Latency	Decoding algorithm	Reference
	Recording	Stimulation				
Cognitive & physiological state monitoring	EEG	N/A	ACC 90% (2‐class)	N/A	CSP^a)^	[221]
Monitoring vigilance/drowsiness	EEG	N/A	ACC 93% (2‐class)	10 s input window	SVM	[222]
Real‐time sleep staging monitoring	EEG	N/A	ACC 95.5% (5‐class)	N/A	LSTM	[335]
Attention state monitoring and classification	EEG	N/A	ACC 81.16% (2‐class)	0.5 s input window	Echo state network	[336]
Mental workload estimation	EEG, EOG	N/A	Pearson's correlation coefficient 0.89 (4‐class)	2 s input window	Random forest model	[337]
Sleep quality and apnea assessment	EEG, EOG, EMG	N/A	ACC 88.52% (sleep apnea detection)	30 s input window	CNN	[338]
Management of comprehensive mental states	EEG, EOG, PPG^b)^	N/A	ACC 92.49% (drowsiness), 92.27% (stress), 84.92% (sleep stages)	N/A	SVM	[339]

^a^ CSP, common spatial pattern;

^b^ PPG, photoplethysmography

A lightweight cognitive BCI has been demonstrated through an ultrathin electronic tattoo‐based skin interface (Figure 8G) [337]. This wireless system employs stretchable electrodes and personalized electrode layouts to stably acquire frontal EEG and electrooculography (EOG) signals, enabling continuous estimation of mental workload during working memory tasks while preserving spectral characteristics and SNR comparable to conventional scalp EEG. The serpentine electrode structure minimizes motion artifacts and noise while maintaining stable signal acquisition during dynamic activities such as walking and running. In addition, the fully integrated system remains lightweight, with a total weight of 8.1 g including a 150‐mAh battery, supporting prolonged wear.

Building on such advances in conformal electrophysiological sensing, cognitive monitoring has been extended toward long‐term assessment in home environments through wireless soft patch‐based sleep monitoring systems integrating EEG, EOG, and EMG (Figure 8H) [338]. These systems maintain stable skin contact and high signal quality during overnight recordings, showing strong agreement with clinical polysomnography (PSG) in sleep staging. Their suitability for extended home use is further supported by representative 7‐day measurements with minimal SNR variation and by negligible resistance change after 1000 repeated stretching cycles. Furthermore, end‐to‐end CNN‐based pipelines enable automated classification of sleep stages and sleep apnea events, demonstrating the feasibility of sustained, at‐home cognitive and physiological monitoring without continuous supervision.

Recent studies extend cognitive state monitoring toward multimodal bioelectronic sensing systems that integrate electrophysiological and physiological signals to enable robust and practical real‐time monitoring. A representative example presents a wireless, soft, forehead‐mounted biopatch that combines these modalities and, through deep‐learning‐based analysis, enables unified classification of fatigue, stress, and sleep states (Figure 8I) [339]. With online mobile signal processing and automated classification, the system supports continuous monitoring and real‐time management of fatigue–stress–sleep feedback loops. The conformal, reusable design minimizes motion artifacts while maintaining clinical‐grade signal quality and long‐term wearing comfort, supporting continuous home‐based cognitive monitoring and personalized digital therapeutics for mental health and sleep disorders.

Overall, recent advances in cognitive state monitoring BCIs highlight a transition from controlled, task‐specific decoding toward continuous and real‐world monitoring enabled by wearable, conformal, and multimodal sensing technologies. Improvements in signal stability, form‐factor design, and on‐device or mobile processing have enabled sustained operation, user comfort, and practical deployment in everyday environments. At the same time, future work should further extend current demonstrations to larger populations and longer‐term studies, while improving robustness under diverse real‐world conditions and ensuring reliable integration of multimodal data for personalized and adaptive cognitive health management.

### 7.4 Entertainment and Creative Applications of BCIs

As an emerging and exploratory direction, BCIs are expanding beyond laboratory and clinical environments into entertainment and creative applications, thereby broadening their practical relevance in everyday life. Entertainment‐oriented BCIs have expanded toward interactive systems that utilize neural signals not only for input but also for monitoring and expressive feedback, particularly in games, VR/AR, and interactive art [340, 341, 342]. Prior studies have demonstrated the feasibility of using EEG‐based signals for game control, emotion and immersion monitoring, and creative expression, with VR/AR integration further enhancing attention and affective engagement [343, 344, 345]. In this context, neural signals can enable more intuitive, personalized, and adaptive interaction with digital environments than conventional interfaces. Importantly, these developments are relevant not only to entertainment itself but also to rehabilitation and broader technological translation. Gamified BCI paradigms can enhance user motivation and engagement in rehabilitation settings, while scalable validation in healthy users can contribute to accelerating technological maturation. However, many of these efforts remain short‐term or proof‐of‐concept, and further validation is required to establish real‐time interaction, continuous control, and signal stability during prolonged use in realistic entertainment environments.

Recent work has increasingly focused on addressing these limitations by enabling continuous and meaningful neural interaction (Table 6). For example, affective BCIs have been developed to estimate users’ arousal and emotional valence from EEG in real time and to dynamically generate musical attributes such as melody and rhythm [346]. By incorporating closed‐loop feedback in which neural responses to the generated content are continuously monitored, these systems demonstrate how entertainment BCIs can support emotion‐responsive content generation and sustained user engagement. In parallel, visual tracking BCIs that spatially encode visual stimuli and translate EEG correlation patterns into continuous velocity vectors have enabled smooth cursor or object tracking (Figure 8J) [347]. Subject‐specific projection matrices further allow personalized interaction by accounting for inter‐subject variability in neural responses. Performance evaluation shows that the achieved Fitt's information transfer rate (ITR) in both fixed and random tracking tasks is comparable to that of related invasive studies, indicating competitive control performance. Demonstrations in free‐form drawing and game‐like tasks illustrate the applicability of BCIs to real‐time entertainment interactions such as painting and gaming.

**TABLE 6:** Applications of BCIs in entertainment and creative interaction.

Task	Signal modality		Performance	Latency	Decoding algorithm	Reference
	Recording	Stimulation				
Hand‐free control for AR interface	EEG	Intent recognition for AR function	ACC 96.4% (3‐class)	N/A	N/A	[158]
Finger decoding and quadcopter game control	Utah MEA	Virtual hand	Completed trials 98.7% (4D decoder)	1.58 s input window (4D decoder)	TRCA^a)^	[274]
VR‐based BCI for text spelling/navigation	EEG	VR display	ACC 91.73% (33‐class)	0.8–2 s input window	CNN	[341]
MI^b)^ with VR training and real‐time control	EEG	VR display	ACC 93% (4‐class)	1–4 s input window	CNN	[342]
3D motor decoding (game, cursor control)	ECoG	Visual feedback	Pearson correlation coefficient 0.84	100 ms input window	LSTM + Kalman filter	[348]
Closed‐loop EEG‐driven emotional music therapy	EEG	Music	ACC 92.5% (4‐class), 95.3% (2‐class)	< 500 ms	CNN + LSTM	[346]
Continuous visual tracking	EEG	Feedback on the time cost	Success rate 92.6% (fixed tracking task)	1 s input window	TRCA	[347]

^a^ TRCA, task‐related component analysis;

^b^ MI, motor imagery

Advances in signal quality and long‐term stability have enabled more sophisticated real‐time entertainment interactions. Studies employing high‐density, flexible µECoG arrays have achieved stable, high‐resolution neural recordings over extended periods and used these signals to decode three‐dimensional limb movements or cursor trajectories in real time for game control (Figure 8K) [348]. Notably, long‐term in vivo evaluations over 203 days show minimal electrode yield degradation (5.49%) and stable SNR above 20 dB, supporting the chronic stability of these systems. Furthermore, intracortical BCIs have enabled continuous decoding of finger‐level motor intentions to support multi‐degree‐of‐freedom game control, allowing users to perform complex movements naturally within virtual environments (Figure 8L) [274]. Such developments highlight the broader applicability of entertainment‐oriented BCIs, including their potential to support social interaction and leisure activities for individuals with SCI. These systems enable naturalistic interaction with minimal training and validate real‐time responsiveness and stability, indicating a transition from conceptual demonstrations toward more practical and sustained real‐world BCI interaction scenarios.

## 8 Conclusion and Perspectives

Current BCI systems continue to advance toward portable, closed‐loop operation, extending neural sensing and interaction beyond laboratory and clinical environments into daily‐use scenarios [349, 350]. Across the works reviewed here, a common trajectory is the transition from proof‐of‐concept decoders evaluated in controlled settings to integrated platforms that acquire signals, perform real‐time inference, and deliver feedback under practical constraints such as motion, power, and long‐term wear. From a systems perspective, this progress is enabled by cross‐layer co‐design spanning (i) flexible bioelectronic interfaces that define electrode–tissue coupling and artifact susceptibility, (ii) miniaturized front‐end electronics and power management that bound noise and operating lifetime, and (iii) wireless telemetry architectures that determine achievable latency and throughput in wearable and implantable form factors. Improvements in electrode materials and conformal mechanics increase comfort and contact stability while reducing motion‐induced artifacts, providing a more reliable sensing substrate for longitudinal operation. At the system level, miniaturized acquisition front ends, efficient power management, and low‐power communication enable low‐latency operation in wearable and implantable form factors. Building on this hardware stack, machine‐learning and deep‐learning approaches have become central to translating high‐dimensional, noisy neural signals into actionable outputs by learning robust spatiotemporal features that generalize across users and sessions, enabling rapid, or in some cases, calibration‐free operation and signaling a shift toward full‐stack BCI co‐design rather than isolated advances.

This full stack development also clarifies why similar architectural principles recur across diverse application domains. Sensory restoration and substitution require stable stimulation interfaces and encoding strategies that can deliver reproducible percepts under chronic use. Assistive communication and motor control demand responsive, low‐latency decoding and reliable interaction loops during natural behavior. Cognitive monitoring and rehabilitation depend on unobtrusive long‐term sensing and the ability to estimate state markers under daily‐life variability to support adaptive intervention. Emerging entertainment and creative‐interaction demonstrations further emphasize that low‐burden form factors and dependable real‐time decoding are central when operation occurs outside controlled environments. Collectively, these examples suggest that the primary drivers of impact will be system‐level decisions that improve long‐term robustness, usability, and closed‐loop latency, rather than incremental gains in offline decoding accuracy alone.

Looking forward, several directions are likely to shape next‐generation BCI platforms. First, deeper integration of flexible interfaces with embedded intelligence will reduce dependence on external computation and high bandwidth streaming by shifting feature extraction and inference onto wearable or implantable hardware. Advances in low‐power processors, neuromorphic/accelerator architectures, and model compression are expected to expand the class of decoders that can run continuously within strict power and thermal budgets. Second, tighter coupling between sensing hardware and learning algorithms will support online adaptation to potential issues such as electrode drift, physiological variation, and motion artifacts, enabling sustained performance. Third, closed‐loop designs are expected to move beyond fixed feedback or stimulation rules toward adaptive control strategies that update intervention parameters based on neural biomarkers and behavioral outcomes in real time. Together, these developments position BCIs not only as neural decoders, but as integrated neuroelectronic systems that co‐optimize interface, computation, communication, and feedback for long‐term operation in everyday settings.

## Conflicts of Interest

The authors declare no conflicts of interest.

## Data Availability

The authors have nothing to report.
